# Aphid-mediated beet yellows virus transmission initiates proviral gene deregulation in sugar beet at early stages of infection

**DOI:** 10.1371/journal.pone.0311368

**Published:** 2024-10-01

**Authors:** Roxana Hossain, Glenda Willems, Niels Wynant, Simon Borgolte, Kristof Govaerts, Mark Varrelmann

**Affiliations:** 1 Department of Phytopathology, Institute of Sugar Beet Research, Göttingen, Lower–Saxony, Germany; 2 Department Genomics and Biotechnologies, SESVanderHave SE, Flemish Brabant, Tienen, Belgium; 3 Department Biotic Stress Management, SESVanderHave SE, Flemish Brabant, Tienen, Belgium; University of Agriculture Faisalabad, PAKISTAN

## Abstract

Beet yellows virus (BYV), one of the causal agents of virus yellows (VY) disease in sugar beet (*Beta vulgaris* subsp. *vulgaris*), induces economically important damage to the sugar production in Europe. In the absence of effective natural resistance traits, a deeper understanding of molecular reactions in plants to virus infection is required. In this study, the transcriptional modifications in a BYV susceptible sugar beet genotype following aphid-mediated inoculation on mature leaves were studied at three early infection stages [6, 24 and 72 hours post inoculation (hpi)] using RNA sequencing libraries. On average, 93% of the transcripts could be mapped to the *B*. *vulgaris* reference genome RefBeet-1.2.2. In total, 588 differentially expressed genes (DEGs) were identified across the three infection stages. Of these, 370 were up- regulated and 218 down-regulated when individually compared to mock-aphid inoculated leaf samples at the same time point, thereby eliminating the effect of aphid feeding itself. Using MapMan ontology for categorisation of sugar beet transcripts, early differential gene expression identified importance of the BIN categories “enzyme classification”, “RNA biosynthesis”, “cell wall organisation” and “phytohormone action”. A particularly high transcriptional change was found for diverse transcription factors, cell wall regulating proteins, signalling peptides and transporter proteins. 28 DEGs being important in “nutrient uptake”, “lipid metabolism”, “phytohormone action”, “protein homeostasis” and “solute transport”, were represented at more than one infection stage. The RT-qPCR validation of thirteen selected transcripts confirmed that BYV is down-regulating chloroplast-related genes 72 hpi, putatively already paving the way for the induction of yellowing symptoms characteristic for the disease. Our study provides deeper insight into the early interaction between BYV and the economically important crop plant sugar beet and opens up the possibility of using the knowledge of identified proviral plant factors as well as plant defense-related factors for resistance breeding.

## 1. Introduction

One of the economically most important viral diseases in European sugar beet (*Beta vulgaris* subsp. *vulgaris*) cultivation is the virus yellows (VY) disease, which is caused by a complex of different aphid-transmissible virus species. Beet yellows virus (BYV), beet mild yellowing virus (BMYV) and beet chlorosis virus (BChV) are most common, while beet mosaic virus (BtMV) has a minor role [1].

Beet yellows virus (BYV), type member of the genus *Closterovirus* in the family *Closteroviridae*, is considered the most devastating pathogen of the YV complex posing a serious threat to sugar beet production and causing yield losses of up to 47% depending on environmental conditions and timing of the infection [2]. In addition to leaf yellowing, typical BYV symptoms are reddish or brownish necrotic spots [1, 3], making this virus well distinguishable from the other sugar beet yellowing viruses in early stages of the disease. The molecular mechanism by which BYV induces symptoms and subsequently reduces root yield and sugar content is still unknown. However, it has been shown that reduced green leaf cover in infected crops leads to a decrease in net photosynthetic rate, restricting sugar beet growth [4]. Moreover, infected plants have smaller leaves and less leaf area compared to healthy plants [3]. BYV is mainly restricted to the phloem, thus virus particles can be found in both parenchyma cells and mature sieve elements. However, the phloem-limitation is incomplete as virions were also detected in vein neighbouring mesophyll cells [5]. The virus can be transmitted to more than 150 plant species, most prominently sugar beet, table beet, Swiss chard and spinach, by the principle insect vectors, namely the green peach aphid (*Myzus persicae* Sulzer) and the black bean aphid (*Aphis fabae* Scopoli), in a semipersistent manner [6–10]. BYV is a filamentous positive-sense single-stranded RNA (ssRNA) virus with a large monopartite genome of approximately 15.5 kb containing nine open reading frames (ORFs) with unique molecular organisation and particle structure [8, 11–14].

VY is currently controlled by growing practices and by the application of foliar insecticides directed against the aphid vector. Nevertheless, these practices are not as effective as the previously used neonicotinoide seed treatment, which was banned in 2018. Cultivars with effective resistance against BYV, BMYV or BChV are not yet available on the market, although breeding efforts have been made in Europe since 1944 to identify resistance against VY, and especially BYV [15]. Quantitative trait loci (QTLs) mapped on chromosomes III, V and VI were reported for BYV [16] as well as a locus controlling vein-clearing symptoms of BYV infection mapped to chromosome IV [17]. A similar breeding program initiated in California in 1955 resulted in two sugar beet hybrids for commercial use. However, these hybrids turned out to be only moderately BYV resistant, and were more effected by aphid probing behaviour and by infestation pressure [15]. Thus, this type of resistance is not sufficient to control the disease.

A crucial step to identify putative breeding targets is a better molecular understanding of the compatible interaction between the virus and its host plant. In plant-virus interactions, host factors can have antiviral (in tolerant/resistant genotypes) or proviral activities (in susceptible genotypes) [18]. Host factors with proviral activity have been identified for all stages of the virus infection cycle: viral RNA translation, viral replication, accumulation, virus movement, and virion assembly. These so-called susceptibility factors could be targets for generating resistance to plant viruses [18].

Since the release of the sugar beet genomic information [19] and the availability of affordable RNA next generation sequencing (NGS), the expression of differentially regulated genes being active during virus infection in plants can be functionally described [20]. Examples in the literature dealing with the production of RNA sequencing (RNA-Seq) transcript libraries and their use to study gene expression induced by altering environmental conditions [21] are already available for other plant-virus interactions covering important crop diseases. Only a few of these studies focus on gene deregulation at the very early stages of infection, and the use of the natural vector for virus transmission to depict the natural initial infection. The early infection phase is therefore important, as it lays the foundation for successful virus establishment. To identify putative breeding targets, it is important to understand which plant genes/proteins favour or are responsible for the replication and spread of the virus during the compatible interaction.

The response of *B*. *vulgaris* to beet necrotic yellow vein virus (BNYVV), transmitted by the plasmodiophorid *Polymyxa betae*, was published recently [22–25]. The transcriptional response of aphid-transmitted viruses has not yet been investigated in *B*. *vulgaris*. To the best of our knowledge, host factors involved in the BYV–sugar beet interaction are still unknown.

By investigating early stages of BYV infection, our study contributes to understand which host genes may act proviral during viral establishment in the plant cell to understand how the initiation of virus infection might be prevented. By using RNA-Seq transcript information from the response induced by natural aphid-mediated transmission of BYV in a susceptible sugar beet genotype, we identified potential susceptibility factors in plants critical to the early BYV-sugar beet interaction. These findings will be fundamental to support and accelerate breeding processes by identifying natural variation in plant factors that lead to incompatibility between sugar beet and BYV. Moreover, the genes identified are potential targets for novel precision breeding techniques, such as CRISPR/Cas, to support breeding for VY resistance in sugar beet in the near future.

## 2. Materials and methods

### 2.1 Plant, virus and vector source, inoculation and sampling

A BYV susceptible sugar beet genotype was supplied by SESVanderHave (SV79). The natural BYV isolate (supplied by Mark Stevens, BBRO UK) was maintained on sugar beet cv. Vasco (SESVanderHave, Tienen), serving as virus source plant. A healthy aphid population of *Myzus persicae* (supplied by the Institute of Horticultural Production Systems, Department Phytomedicine, Leibniz University Hannover) was reared on healthy sugar beet cv. Vasco. SV79 seeds were sown in peat soil and seedlings were transplanted to individual pots 7 days after germination. Plants were cultivated at (22–25 ° C, humidity 75-85%, photoperiod of 16/8 h light/dark, daily watering, and nutrient supply via universal fertilizer every two weeks). Fourteen days after transplanting (4–6 leaf stage), two leaves per plant and in each treatment a total of 24 plants were inoculated either with BYV-viruliferous (BYV-inoculation) or with healthy *M*. *persicae* (mock-inoculation). For virus acquisition, healthy aphids were allowed to feed on the source plant for three days. For virus inoculation, five wingless aphids per leaf were used and the leaf was covered with an organza bag tied at the stem to prevent aphid escape. For the mock-control, aphids that had fed on a healthy sugar beet for three days, were transferred to the test plant. Leaf samples were taken at 6, 24 and 72 hours post inoculation (hpi) in four replicates per treatment. One leaf per plant was cut off, organza bags removed, and a 100 mg piece of leaf was excised at the aphid feeding position (point of initial infection), which was subsequently eliminated by hand. The leaf piece was put into a 2 mL tube (Eppendorf SE, Hamburg), immediately frozen in liquid nitrogen and stored at—70°C until further processing. The second organza bag was removed after the final sampling time point, and all plants were treated with the systemic insecticide Teppeki (Belchim Crop Protection Deutschland GmbH, Burgdorf) and observed for further six weeks for symptom documentation and virus detection by DAS-ELISA [26] using BYV specific antibodies and protocols provided by the manufacturer (DSMZ, Braunschweig).

### 2.2 RNA extraction and sequencing

100 mg leaf tissue was ground to fine powder in liquid nitrogen in the 2 mL tube using a pistil. Total RNA was extracted using the Direct-zol^™^ RNA Miniprep Kit (Zymo Research Europe GmbH, Freiburg) according to the manufacturer’s instructions. RNA was checked for quantity and quality using a spectrophotometer (DeNovix DS-11, Wilmington) and gel electrophoresis. 20 μL (≥ 20 ng/ μL, OD260/280 ≥ 2.0; OD260/230 ≥ 2.0) before transcriptome sequencing. The remaining RNA was stored at—70°C for further RT-PCR and RT-qPCR analysis. A commercial sequencing service (Novogene (UK) Company Limited, Cambridge) prepared cDNA libraries after eukaryotic mRNA library poly A enrichment. Library quality was controlled prior to sequencing with the NovaSeq PE150 sequencing platform (Illumina High Throughput Sequencing, 9 G of raw data per sample). After data quality control, sequencing results were delivered.

### 2.3 Transcriptome analysis and figure preparation

For comparative transcriptome data analysis, CLC Genomics workbench (Qiagen, Aarhus) was used. Data from each of the four replicates per treatment (BYV-inoculated and mock-inoculated) were mapped to the sugar beet reference genome RefBeet-1.2.2. A principal component analysis (PCA) was performed to check for clustering of replicate samples. Subsequently, the BYV- and mock-inoculated treatments at 6, 24 and 72 hpi were compared to generate a list of differentially expressed genes (DEGs). Genes with a minimum fold change of 1.5, and false discovery rate (FDR) adjusted p- values below 0.05 were classified as significantly expressed genes. For further analysis, MapMan application (MapMan 3.6.0RC1, hosted at Forschungszentrum Jülich), which was designed to cover plant-specific pathways and processes was used [27, 28]. Therefore, a table was prepared containing all log_2_ fold change data of the DEGs at the different infection stages according to 1) FDR- adjusted p- value < 0.05 and 2) fold changes > 1.5. Data were assigned "0" if not significant and "X" if not available. A reference mapping file for *B*. *vulgaris* was downloaded from https://mapman.gabipd.org/mapmanstore and imported into MapMan to perform Venn diagram generation, gene annotation and gene category analysis. Figures and statistics were generated with CLC Genomics Workbench, R (version 4.2.0, R Foundation for Statistical Computing, Vienna), and Microsoft Excel (Microsoft Corporation, Redmond).

### 2.4 RT-PCR and RT-qPCR

The backup RNA samples were used for cDNA-synthesis (300 ng RNA) using SuperScript^™^ IV Reverse Transcriptase (Thermo Fisher Scientific Inc., Waltham) according to the manufacturer’s instructions. Virus detection was performed for BYV- and mock-inoculated samples for all time points and replicates using BYV specific primers BYV -s3 5 ´GTTAACACAGTTACTAAGGTTCCA´ 3 and BYV -as2 5 ´TGGAGGTATACCAAAGGAAGTTCA´ 3 based on GenBank accession no. NC_001598 (personal communication Wulf Menzel, DSMZ, Braunschweig).

The expression of thirteen *B*. *vulgaris* genes was measured by RT-qPCR to validate the transcriptomic data. The genes were manually selected to cover the range of up- and down regulated genes at either one or more than one time point, focusing partly on chloroplast-related genes. Primers for target genes (S1 Table) were designed using Primer-BLAST. Sugar beet glyceraldehyde-3-phosphate-dehydrogenase and elongation factor 2 were used as reference genes. To show that expression deregulation is RNA sample independent, another set of leaf samples from the same experiment was used. Total RNA was extracted from four different biological replicates as described above, and 300 ng of total RNA was reverse transcribed into cDNA. The cDNA was diluted 1:9 in water prior to its application in RT-qPCR. The reaction was set up in 20 μL volume containing 2× iTaq Universal SYBR Supermix (BioRad, Feldkirchen, Germany), 400 nM of each primer and 2 μL cDNA. The RT-qPCR was carried out in the CFX96 Real Time System C1000 Touch Thermal Cycler (Bio-Rad, Feldkirchen, Germany). The reaction was set with initial denaturation at 95°C for 3 min. followed by 40 cycles at 95 °C for 30 s, 58 °C for 20 s, 72 °C for 30 s, and final extension at 72 °C for 5 min. Each biological sample was analysed in two technical replicates. Data normalization and calculation of relative expression values was done using the 2−ΔΔCt method [29].

## 3. Results

### 3.1 Bioassay

**Fig 1:**
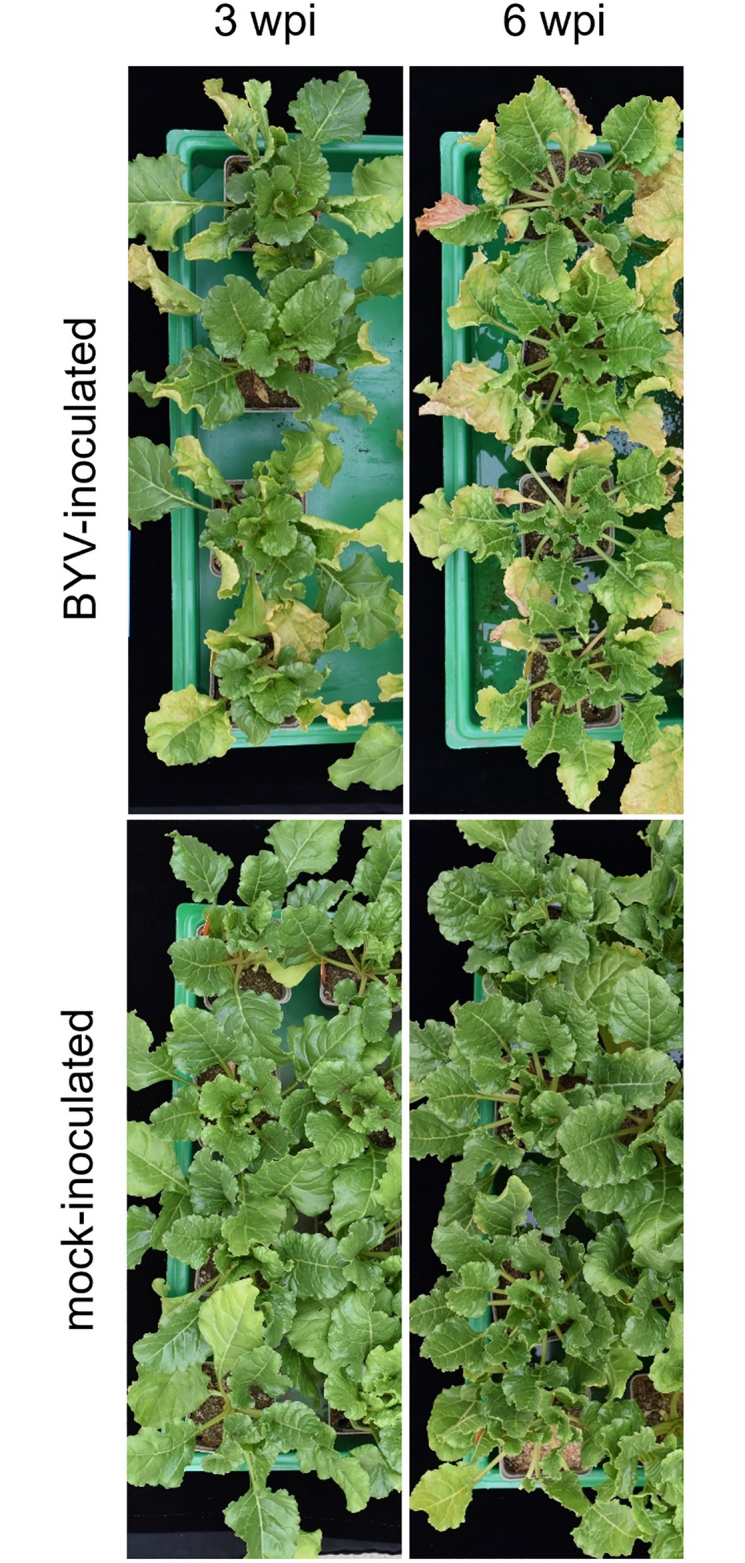
Beet yellows virus (BYV) induced symptoms in *B*. *vulgaris* cultivar SV79 three and six weeks after aphid inoculation (wpi) compared to mock-inoculated SV79 sugar beet plants.

After the final leaf sampling for RNA-Seq at 72 hpi, plants were further cultivated in a climate chamber and rated for symptom expression. All the BYV-inoculated plants developed typical symptom expression, namely yellowing on the leaf tip or margins and reddish necrotic spots two to three weeks after inoculation. Leaves of mock-inoculated plants stayed healthy and green (Fig 1). DAS-ELISA test showed that 100% (24/24) of the BYV-inoculated plants were infected, while no absorbance (OD at 405 nm) was measured in samples of the mock-inoculated plants. Virus detection was performed for all RNA/cDNA samples used for gene expression validation in RT-qPCR by using RT-PCR. BYV was not detectable in 6 and 24 hpi samples. For 72 hpi samples, the detection signal of BYV was very low and not measurable in all the four sample replicates (S1 and S2 Figs).

### 3.2 Sequencing statistics, Principal Component Analysis (PCA)

**Fig 2:**
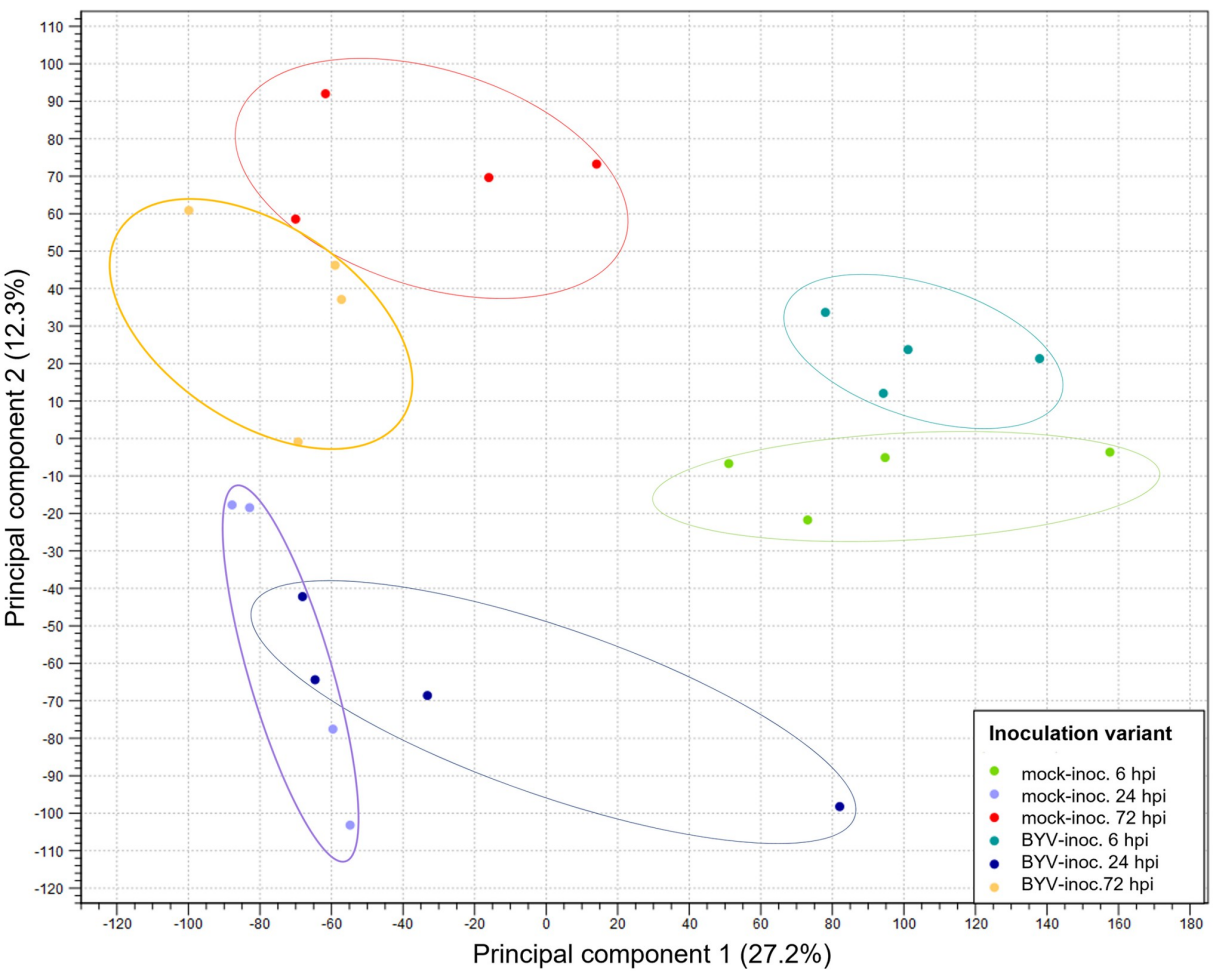
Principal component analysis (PCA) showing clustering of inoculation treatment replicates (n = 4). Light green = mock-inoculated, 6 hours post inoculation (hpi); dark green = BYV-inoculated, 6 hpi; purple = mock-inoculated, 24 hpi; blue = BYV-inoculated, 24 hpi; red = mock-inoculated, 72 hpi; yellow = BYV-inoculated 72 hpi.

Between 59 and 98 million raw reads of good quality (filtered reads after data quality analysis were > 98%) were obtained for the 24 RNA samples that were sequenced. 92-95% of the filtered raw reads could be mapped in pairs to the sugar beet reference genome RefBeet-1.2.2 (Table 1) covering approximately 97% of the 26,569 genes and 28,721 transcripts, respectively. Reads mapped in broken pairs ranged from 3.87 to 5.87%. Reads that could not be mapped were detected at low percentage (1.06–2.85%) (S2 Table). The PCA of the 24 samples, proved that samples combined into principal clusters, showing similarities between the sample replicates, and distinguishing inoculation treatments and sampling time points (Fig 2). The inoculation treatments (mock-inoculated and BYV-inoculated) differed from each other. The difference became even greater when different time points (6, 24, 72 hpi) were compared. Only at 24 hpi, there were sample overlaps between two clusters.

**Table 1:** Summary of raw Illumina sequencing reads after sequence quality check, raw data filtering, and alignment of the reads to the *B*. *vulgaris* reference genome RefBeet-1.2.2 for beet yellows virus (BYV)-inoculated and mock (healthy aphids)-inoculated sugar beet leaf samples.

Inoculation	Sampling [hours post inoculation]	Replicate	Raw reads	Filtered reads [%]	Reads mapped in pairs to B. vulgaris [%]
BYV	6	1	59,323,324	99.19	93.35
		2	68,364,946	99.22	93.21
		3	83,679,302	99.34	94.27
		4	76,257,018	99.34	95.07
	24	1	60,113,316	99.26	94.24
		2	60,715,156	99.28	93.38
		3	71,679,552	99.34	93.87
		4	76,398,520	99.24	93.7
	72	1	61,247,066	99.00	92.8
		2	60,696,002	99.10	92.8
		3	61,905,044	99.18	93.21
		4	61,297,456	99.12	92.92
Mock (healthy aphids)	6	1	98,398,464	99.35	94.69
		2	64,809,156	99.29	94.68
		3	71,906,642	99.34	94.17
		4	86,923,492	99.29	93.62
	24	1	69,106,250	99.11	93.07
		2	74,626,200	99.18	93.92
		3	64,873,444	98.90	93.65
		4	62,250,144	99.06	92.28
	72	1	74,606,374	99.05	93.34
		2	62,993,452	98.95	92.72
		3	63,519,148	98.66	92.64
		4	70,460,896	98.96	92.1

### 3.3 Establishment of DEG lists, Volcano plotting

**Fig 3:**
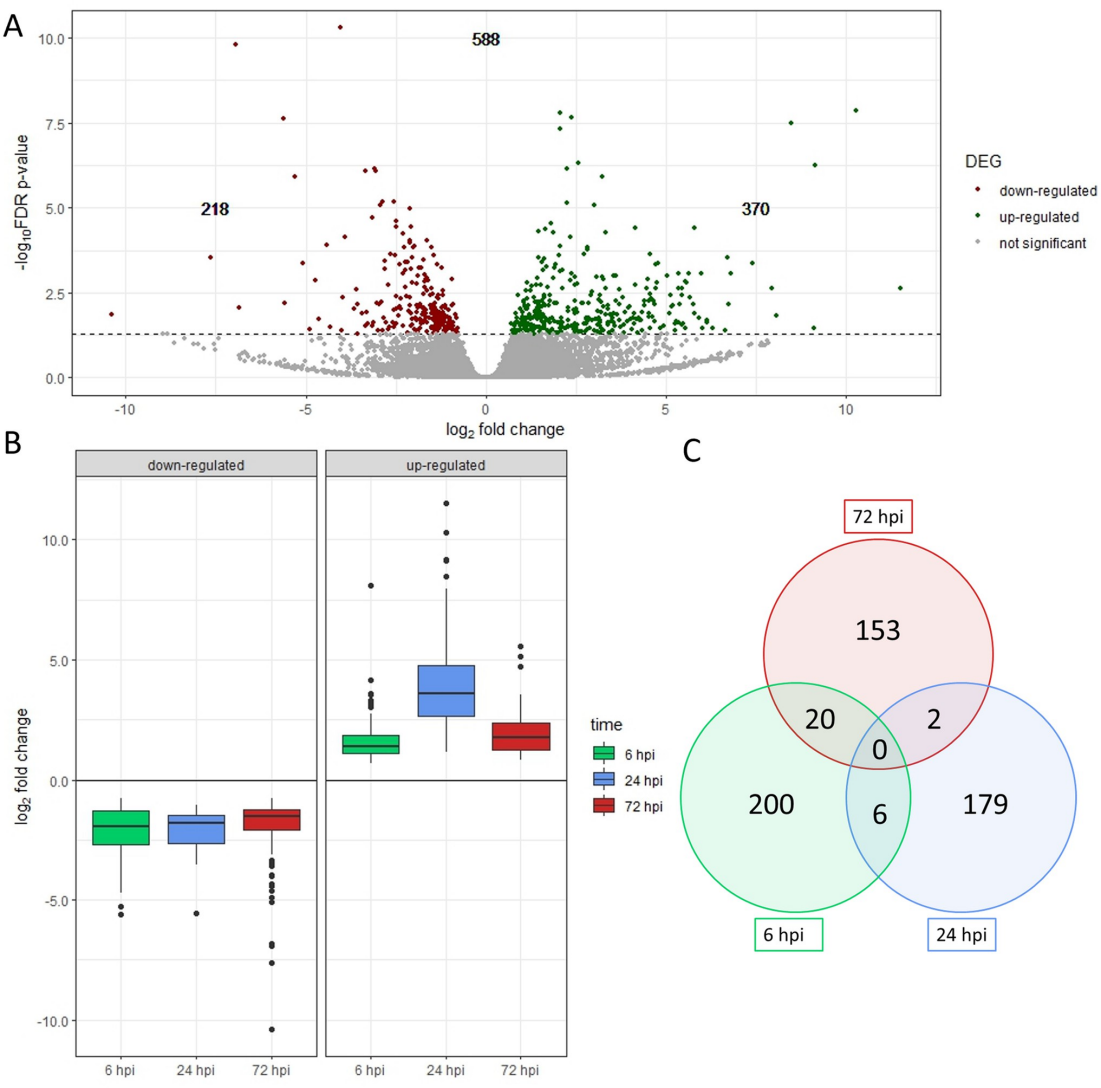
Distribution of differentially expressed genes (DEGs) after BYV infection in sugar beet summarized for all three sampling time points [6, 24 and 72 hours post inoculation (hpi)] shown by the logarithmic representation of p-value and log_2_ fold change using a Volcano plot (A). Boxplots of the log_2_ fold change of DEGs over time at 6, 24, and 72 hpi) (B). Venn diagram analysis comparing DEGs at 6 hpi (green circle), 24 hpi (blue circle), and 72 hpi (red circle) at threshold 0.0. Circle interfaces show, which genes are differentially expressed at two or more of the early infection stages (C).

For each of the samples collected at 6, 24 and 72 hpi, a comparative transcriptome analysis was performed, comparing the BYV-inoculation treatment with the respective mock-inoculation treatment to eliminate the effects of aphid feeding and to filter out the effects of early virus infection. Considering a minimum fold change of 1.5, and FDR- adjusted p- values below 0.05, 588 (equates to ~ 2%) differentially expressed genes (DEGs) were identified from a total of 28,668 genes. On average, 21,477 genes (~ 75%) were not significantly regulated, and data were not available for an average of 6.995 (~ 24%). At 6 hpi, 226 genes (0.79%) were differentially regulated, of which 134 were up- and 92 down-regulated. At 24 hpi, 187 DEGs (0.65%) were identified, 174 genes were up- and 13 were down-regulated. At 72 hpi, 175 DEGs (0.61%) were identified, of which 62 were up- and 113 down-regulated (Table 2). The distribution of significantly and not significantly deregulated genes for all time points is summarized in a Volcano plot (Fig 3A). Fig 3B shows the development over time of the early infection phases and shows that there was a reversal in the up- and down-regulated genes at 6 hpi and 72 hpi. While the genes at 6 hpi are regularly divided between up- and down-regulated genes, at 24 hpi, up-regulated genes clearly dominated, while at 72 hpi there was a shift towards down-regulated genes.

**Table 2:** Number of differentially expressed genes (DEGs) up- and down-regulated at 6, 24 and 72 hours post inoculation (hpi) following aphid-mediated BYV inoculation in sugar beet.

*time point [hpi]*	no. of DEGs		
	Total	Up	Down
*6*	226	134	92
*24*	187	174	13
*72*	175	62	113
***Total***	**588**	**370**	**218**

### 3.4 Venn diagram analysis

A Venn diagram was created using MapMan comparing the different stages of BYV infection to identify matches (Fig 3C).

**Fig 4:**
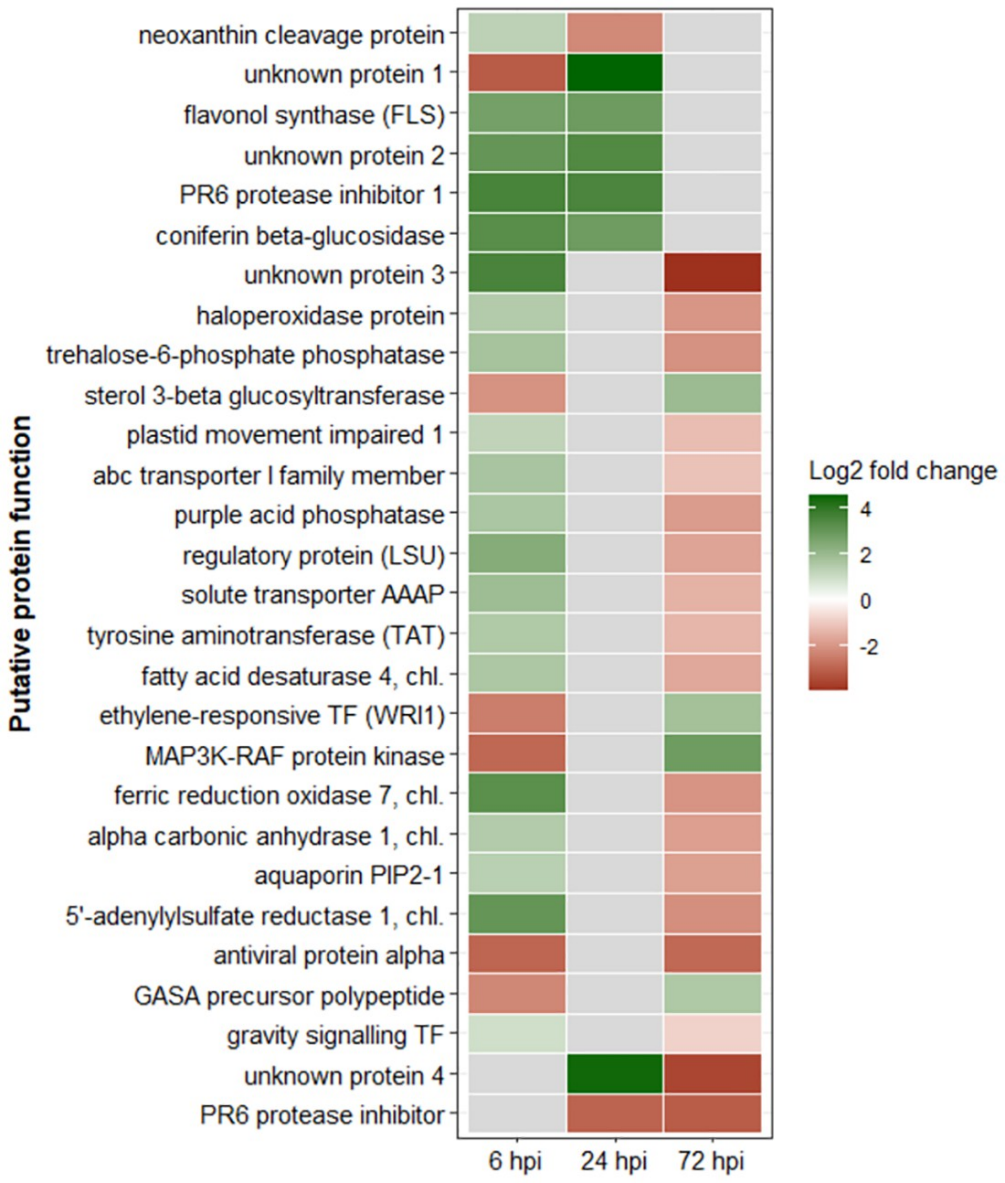
Log_2_ fold change levels of differentially expressed genes (DEGs) with their putative protein function identified within the commonly DEGs that were deregulated at two of the three sampling time points [6, 24 and 72 hours post inoculation (hpi)]. Grey tiles indicate non-significant deregulation.

In total, 28 common genes were identified. Between 6 and 24 hpi, six identical DEGs were identified, of which four were similarly up-regulated, and the remaining two were down- regulated. None of them was significantly regulated at 72 hpi. Between 6 and 72 hpi, twenty identical DEGs were identified, only one of which was similarly regulated (down-regulation), the majority of the DEGs (15) showed up-regulation at 6 hpi and down-regulation at 72 hpi. Four DEGs showed down-regulation at 6 hpi and subsequent up-regulation at 72 hpi. None of these DEGs were significantly regulated at 24 hpi. Only two DEGs were identified by comparing 24 and 72 hpi. One gene was down-regulated at both time points and the other one was up-regulated at 24 hpi and down-regulated at 72 hpi. There were no genes that were differentially regulated at all three infection stages (Fig 4).

### 3.5 DEG categorisation

**Fig 5:**
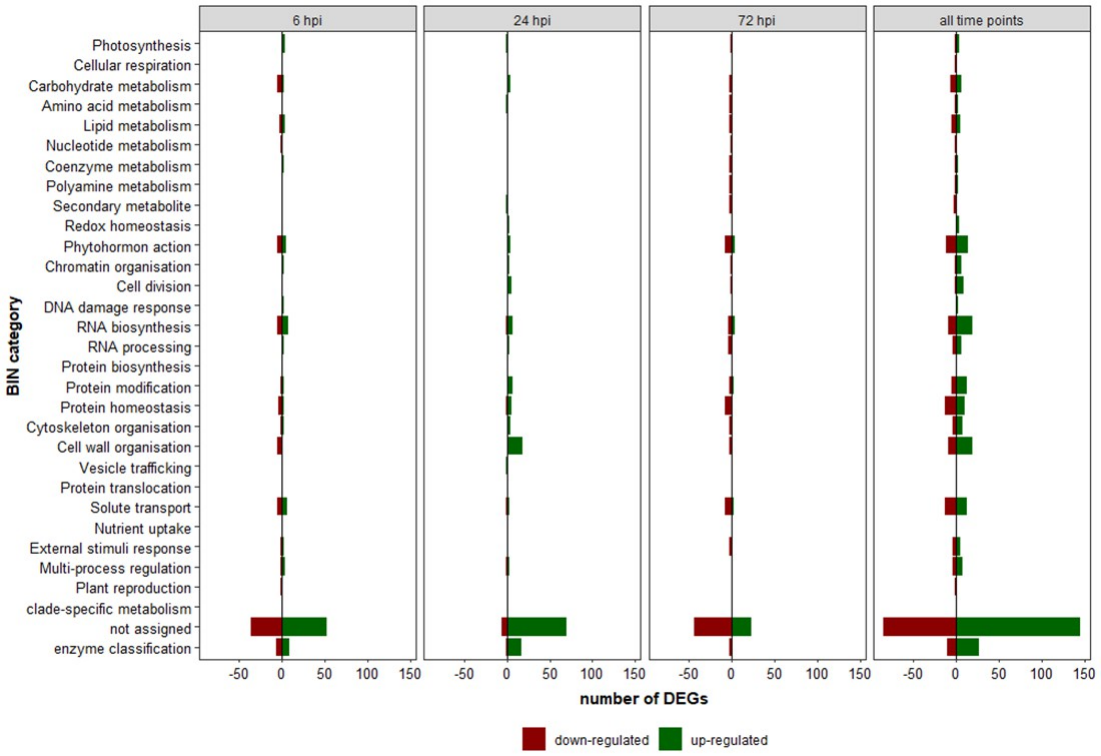
Distribution of up- and down-regulated differentially expressed genes (DEGs) within the different BIN categories at 6, 24 and 72 hours post inoculation (hpi) and combined for all time points.

MapMan major functional categories called "BINs" are rooted into 31 main classes, which are subdivided in subBINs to describe not only the main processes in plants, but also direct assignments to genes with known functions. The DEG list from the three early infection stages was used to assign them to these BINs. Enriched categories could be obtained but also very specific protein functions, if available in the databases used (mercator, prot-scriber, swiss-Prot). Fig 5 summarizes DEG categorisation into the main BINs at 6, 24 and 72 hpi and combined for all time points, showing the distribution of up- and down-regulated DEGs within the different categories. S3 Table shows the exact numbers of DEGs for each time point assigned to the specific BIN category with up- and down-regulated numbers.

**Fig 6:**
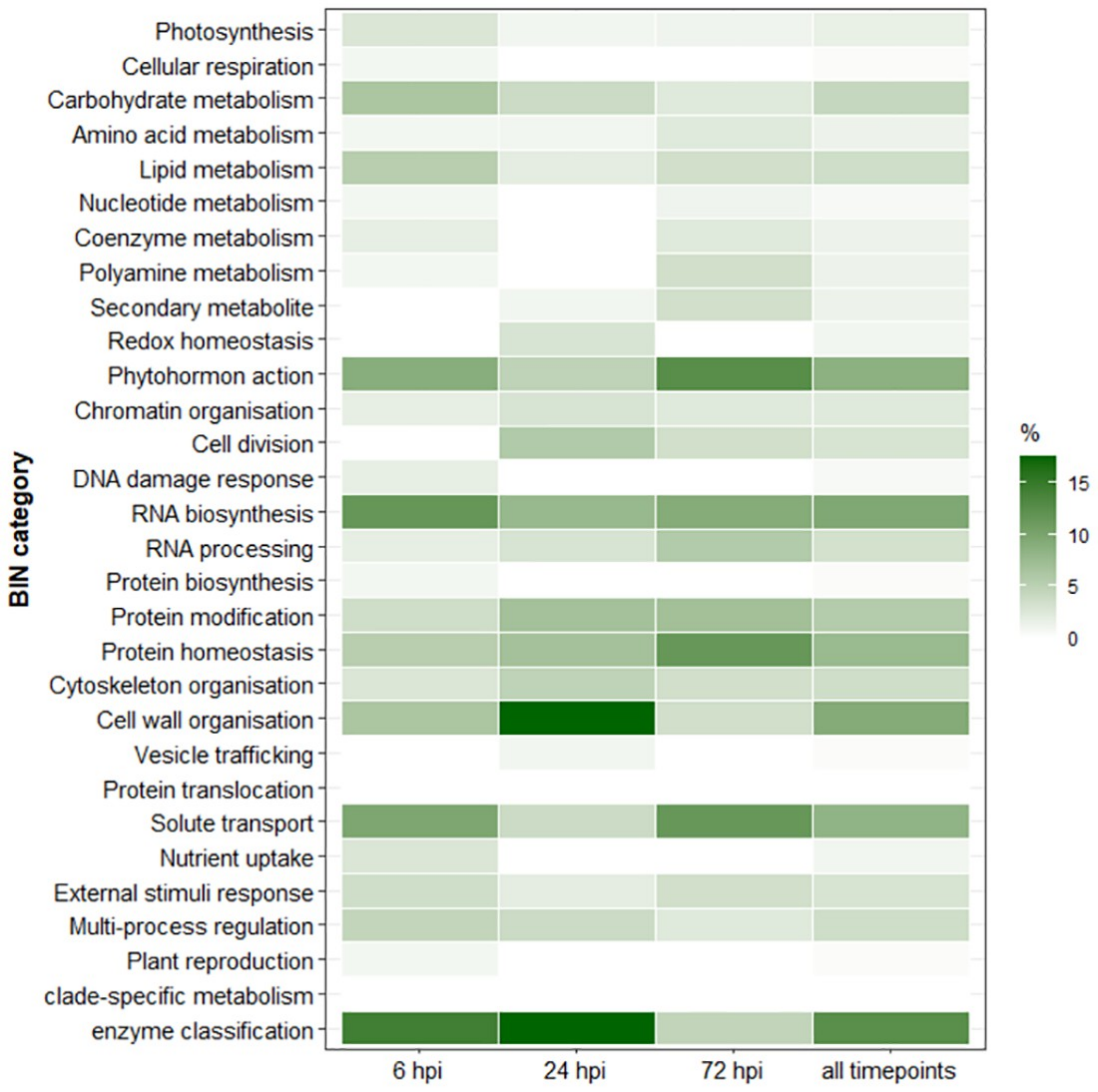
Percentage classification of the BIN functional categories at 6, 24 and 72 hours post inoculation (hpi) and combined for all three time points, adjusted for the category “not assigned”.

For all DEGs, both individually and commonly regulated, the largest proportion of DEGs were in the BIN category “not assigned” (43% and 29%, respectively). Not assigned DEGs were then excluded from the analysis to unravel the most enriched BIN categories with known features. When data from all sampling time points were combined, 29 of the 31 BIN categories were covered. Considering only the commonly regulated DEGs were considered, 15 of the 31 BINs were covered. Fig 6 shows the percentage of enriched categories (excluding “not assigned”) for the individual and for the combined time points.

The top three BIN categories for all infection stages were: “enzyme classification” (13%), “RNA biosynthesis” (10%) and “cell wall organisation” and “phytohormone action” with 9% each. At 6 hpi, DEGs were assigned to 24 different BIN categories, the top three being “enzyme classification” (14%), “RNA biosynthesis” (12%), and “solute transport” (10%). The “enzyme classification” category mainly included oxidoreductases (e.g. cytochrome p450 CYP736A12, ferredoxin nadp reductase isozyme 1, chloroplastic) and glycosyltransferases (e.g xyloglucan endotransglucosylase hydrolase, alpha trehalose phosphate synthase), which were mainly down-regulated. The “RNA biosynthesis” category included transcription factors of DOG-, bHLH-, and ERF-X-type, which were down-regulated and MYB class transcription factors, which were up-regulated. The “solute transport” category included balanced up- and down-regulation with the ABCG and UmamiT (WAT1-related protein) solute transporter down-regulated and the anion transporter and abscisic acid transporter (NRT1/PTR) genes up-regulated.

At 24 hpi, DEGs were assigned to 20 BIN categories, of which 17% were assigned to “cell wall organisation” and “enzyme classification”, 8% to “RNA biosynthesis” and 7% to “protein modification” and “protein homeostasis”, respectively. The “cell wall organisation” category included proteins involved in cellulose modifications (endo-1,4-beta-glucanase), hemicellulose biosynthesis (mannan synthase), pectin modifications (pectinesterase, beta-galactosidase, pectate lyase), expansin activities and lignin, cutin and suberin biosynthesis (lignin peroxidase, cutin synthase), which were consensually highly up-regulated. The BIN category “enzyme classification” included oxidoreductases, acyltransferases, glycosyltransferase, glycosylases, all of which were up-regulated, except a prolyl endopeptidase, which was significantly down-regulated. The “RNA biosynthesis” category included several different transcription factors of the GATA, GRAS-type, zf-HD-type, and AP2-type, which were highly up-regulated except for a transcription factor of the BBX class-V (B-box zinc finger protein 32), which was down-regulated. The “protein modification” category included proteins like extensin protein kinases, LRR-III protein kinases, and for “protein homeostasis” ubiquitin ligase, proteases and protease inhibitors (PR6), all of which were all highly up-regulated, except for PR6, which was highly down-regulated.

At 72 hpi, DEGs covered 21 BIN categories, of which 13% were assigned to “phytohormone action”, 11% to “protein homeostasis” and “solute transport” and 9% to “RNA biosynthesis”. While “phytohormone action” is described in more detail in the next chapter, because of its particular importance in plant defense, “protein homeostasis” and “solute transport” related genes are mentioned here: proteins with chaperone activity (Hsp70, class-C-I small heat-shock-responsive protein; down-regulated), of the ubiquitin-proteasome system (ubiquitin ligase, up- or down-regulated) and protein storage function (Vicilin class-I seed storage protein/antimicrobial peptide, down-regulated) were identified. In the “solute transport” category, anion transporter (NRT1/PTR) were consistently down-regulated, as well as a potassium cation transporter (HAK/KUP/KT) and a fluoride anion export channel (FEX), while there was a highly up-regulation for the solute transporter UmamiT (WAT1-related protein).

Spearman rank correlations of the categories were close between 24 and 72 hpi (rank correlation coefficient ρ = 0.76, p-value = 7.4508E-07), followed by 6 and 24 hpi (ρ = 0.68, p-value = 2.3772E-05) and 6 and 72 hpi (ρ = 0.65, p-value = 7.90986E-05), indicating that similar categories or genes were deregulated at early infection stages.

For the common DEGs, the top three categories were: “nutrient uptake” (15%), “lipid metabolism” (10%) and “phytohormone action” as well as “protein homeostasis” and “solute transport” (all 10%).

Between 6 and 24 hpi, two of the six commonly differentially regulated genes were in the “not assigned” category (one down-regulated at 6 and up-regulated at 24 hpi; one up-regulated at both time points). The remaining four genes were all up-regulated at both time points and were related to the BIN categories “phytohormone action” (neoxanthin cleavage protein), “secondary metabolism” (flavonol synthase FLS), “cell wall organisation” (coniferin beta-glucosidase) and “protein homeostasis” (PR6 protease inhibitor).

Between 24 and 72 hpi, two commonly regulated genes were found, one of which could not be assigned but was highly up-regulated at 24 and down-regulated at 72 hpi, and the other gene could be assigned to “protein homeostasis” (PR6 protease inhibitor, down-regulated at both time points).

**Fig 7:**
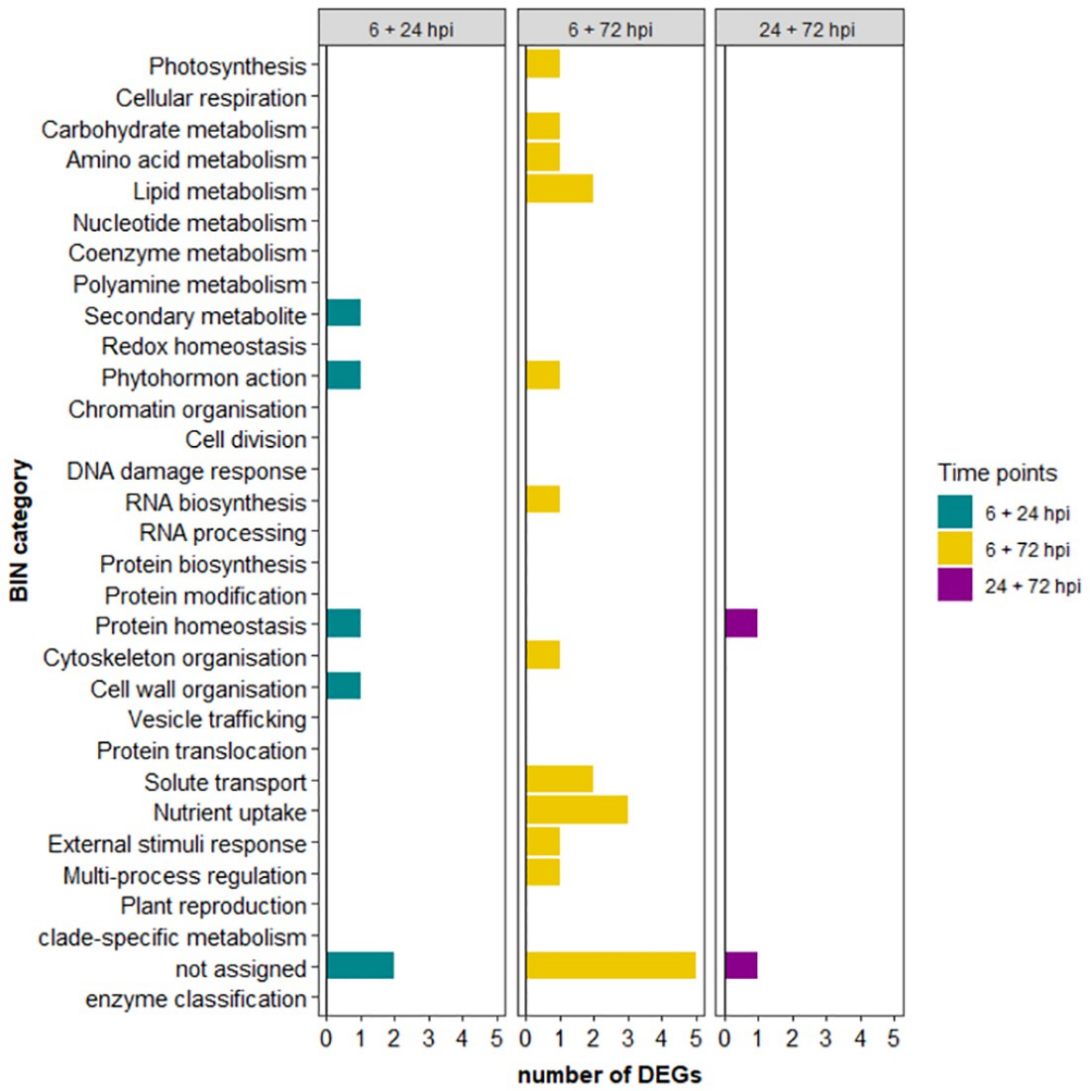
Distribution of common differentially expressed genes (DEGs) between 6 and 24 hours post inoculation (hpi), 6 and 72 hpi and 24 and 72 hpi.

The largest group of commonly regulated genes was identified between 6 and 72 hpi, of which the largest group was “nutrient uptake” with genes up-regulated at 6 and down-regulated at 72 hpi (regulatory protein LSU of sulfate homeostasis, 5’-adenylylsulfate reductase 1, chloroplastic, ferric reduction oxidase 7, chloroplastic). Genes of the BINs “multi-process regulation” (MAP3K-RAF protein kinase), “RNA biosynthesis” (ethylene-responsive transcription factor WRI1), “phytohormone action” (GASA precursor polypeptide) and “lipid metabolism” (sterol 3-beta-glucosyltransferase) were down-regulated at 6 hpi and up-regulated at 72 hpi. Eight other genes were up-regulated at 6 hpi and down-regulated at 72 hpi belonging to “external stimuli response” (gravity signalling transcription factor SCR), “cytoskeleton organisation” (plastid movement impaired 1), “solute transport” (aquaporin PIP2-1), “photosynthesis” (alpha carbonic anhydrase 1, chloroplastic), “amino acid metabolism” (tyrosine aminotransferase TAT), “lipid metabolism” (fatty acid desaturase 4, chloroplastic), “carbohydrate metabolism” (trehalose-6-phosphate phosphatase) and “solute transport” (solute transporter AAAP). Four genes that could be not assigned to a specific BIN were up-regulated at 6 and down-regulated at 72 hpi. One unassigned gene which might act as antiviral protein (swiss-prot annotation) was down-regulated at both time points. The classification and distribution of all identified common DEGs in the various BIN categories is summarised in Fig 7. A detailed listing is given in S4 Table.

### 3.6 BYV effect on phytohormone deregulation

The phytohormone pathway and signaling-related genes were most enriched in our 72 hpi dataset. Salicylic acid (SA), jasmonic acid (JA) and ethylene (Et) are primarily involved in defense mechanisms, with contributions from Auxin (Aux), gibberellins (GA) cytokinins (CK), and abscisic acid (ABA) [30]. The SA and ET pathways were not found to be deregulated in the early BYV infection phase. However, the JA-related gene jasmonic acid oxidase JOX/JAO was down-regulated at 72 hpi. We found two Aux pathway-related genes, the auxin signaling F-Box 3 and the auxin efflux transporter PILS, which were up-regulated at either 24 hpi or 72 hpi. The GA-related gene gibberellin 3-beta-dioxygenase 1 showed down-regulation at 72 hpi. The highest number of deregulated genes was found within the CK pathway. These genes were mainly down-regulated, exception for the cytokinin signal transducer AHP, which was up-regulated at 72 hpi. Two genes, the abscisic acid transporter AIT and the abscisic acid 8’-hydroxylase of the ABA pathway were up-regulated at either 6 or 24 hpi. The 9-cis-epoxycarotenoid dioxygenase NCED1 (chloroplastic) was deregulated at both time points, showing up-regulation at 6 hpi and down-regulation at 24 hpi. Furthermore, 12 signaling peptides were found in our data set, five at 6 hpi (PR-1 and GAST1 up-regulated; two gibberellin-regulated proteins and the GPI-anchored protein LLG1 down-regulated), four at 24 hpi (leucine-rich repeat receptor-like protein kinase PXC2, pathogenesis-related protein 1, RGF precursor polypeptide and EPF-peptide co-receptor TMM up-regulated) and three at 72 hpi (two gibberellin regulated proteins up-regulated and a pythosulfokine precursor polypeptide PSK down-regulated).

### 3.8 Chloroplast-related genes

The most prevalent visible symptom induced by BYV is leaf yellowing or, more specifically chlorosis, reflecting altered pigmentation and structural changes of the chloroplasts. Therefore, we screened our transcriptomic data for significantly deregulated genes related to photosynthesis and chloroplasts. Six photosynthesis-related proteins were identified: a component PsbY of the PS-II complex, a peroxisomal hydroxypyruvate reductase HPR1, a PEP carboxylase PPC, which were up-regulated at 6 hpi, ferredoxin-1, which was up-regulated 24 hpi and an LHC-related protein ELIP, which was down-regulated at 72 hpi. For the commonly regulated DEGs, one alpha-type carbonic anhydrase was identified to be up-regulated at 6 and down-regulated at 72 hpi. In the case of chloroplast-related DEGs, 35 genes were identified, 14 at 6 hpi, four at 24 hpi and 13 at 72 hpi, one commonly regulated at 6 and 24 hpi and four commonly regulated at 6 and 72 hpi. A summarized list of the chloroplast-related DEGs is given in S5 Table.

### 3.9 DEGs with a particularly high transcriptional change

We focused on DEGs that had a log_2_ fold change higher than -5 or 5. At 6 hpi, we found the gibberellin-regulated protein 1 and the probable xyloglucan endotransglucosylase/hydrolase protein 23 with -5.615-fold and -5.294-fold, respectively, with the strongest down-regulation. An uncharacterized protein with gene homology to At5g41620 (TAIR: intracellular protein transporter USO1-like protein, located in the chloroplast) had the highest up-regulation.

At 24 hpi, we identified the PR6 protease inhibitor with the strongest down-regulation of -5.577 (log_2_ fold-change). In the group of up-regulated DEGs, 40 were identified, most of which were cell wall regulating proteins, transcription factors (zf HD-type, A/B-GATA, AP2-type) and signalling peptides. Two DEGs were changed more than 10-log_2_ fold but had unknown protein functions.

At 72 hpi, five DEGs were down-regulated more than -5 (log_2_ fold-change), one vacuolar protein sorting associated protein (-10.398), one transcription regulator (NOT3) protein (-7.623), two proteins with unknown functions (-6.938 and -6.846) and one class-C-II small heat-shock-responsive protein (-5.069). Two DEGs were found to be the most highly up-regulated: the solute transporter UmamiT (5.16) with gene homology to At4g08290 [WAT1-related protein active in the plasma membrane (TAIR)] and the plant cadmium resistance protein (5.546). A list of these genes can be found in S6 Table.

### 3.10 Validation of gene expression

**Fig 8:**
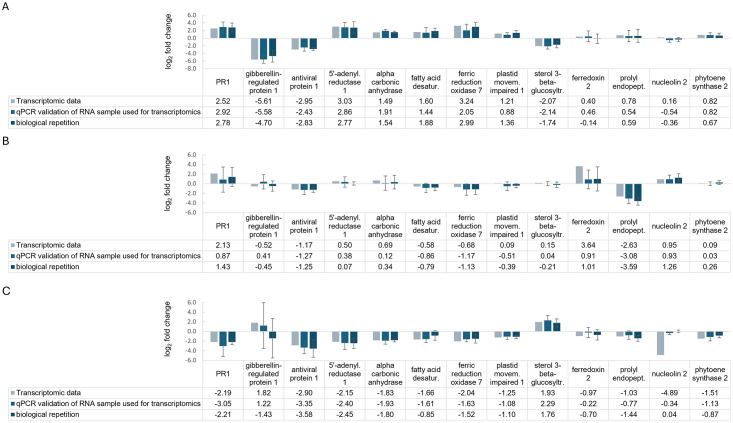
Comparison of log_2_ fold changes for gene expression validation of thirteen selected significantly regulated sugar beet genes 6 (A), 24 (B) and 72 (C) hours after aphid-mediated BYV inoculation in transcriptomic, RT-qPCR validation and in biological repetition samples. Standard deviations are shown for the RT-qPCR validation and the biological repetition samples (n = 4).

Selected genes for gene expression validation and significance levels for RT-qPCR validation and biological repetition samples are shown in S7 Table. Genes were selected to cover gene expression validation in solely expressed genes of the three sampling time points and to cover commonly regulated genes, mostly chloroplast-related. Gene expression was analysed based on the two reference genes elongation factor 2 and glyceraldehyde-3-phosphate dehydrogenase, which showed stable gene expression with and without BYV infection. In general, specific gene regulation could be confirmed for the majority of significantly regulated genes of the transcriptomic analysis (Fig 8).

At 6 hpi, clear RT-qPCR validation of transcriptomic data for all nine significantly deregulated genes was obtained for the transcriptome RNA validation samples and for the biological repetition samples, except for fatty acid desaturase 4, which was not significantly regulated in the transcriptome validation samples. Not significantly regulated genes of the transcriptomic analysis showed the same trends, except for ferredoxin-2 and nucleolin 2, which also showed slightly opposite expression. At 24 hpi, ferredoxin-2 and prolyl endopeptidase were significantly regulated during transcriptomic analysis, which was confirmed by RT-qPCR, but only prolyl endopeptidase was significantly regulated in the validation and replicate samples. As expected, all other genes tested showed high variation in gene expression but followed the same trends. At 72 hpi, significant RT-qPCR validation of the transcriptomic data was obtained for 7/9 significantly regulated genes (exception: nucleolin 2 and phytoene synthase 2). In the biological replicate only five of the tested genes were significantly regulated (antiviral protein I, 5’-adenylylsulfate reductase 1, alpha carbonic anhydrase 1, actin stability factor PMI1 and sterol 3-beta-glucosyltransferase). Prolyl endopeptidase was significantly down-regulated only in the biological repetition samples. For PR-1, which was not significantly down-regulated in the transcriptomic and their validation samples, the biological repetition showed a significant down-regulation. Other genes tested showed high variation in gene expression but also followed the same trends as at 24 hpi.

**Fig 9:**
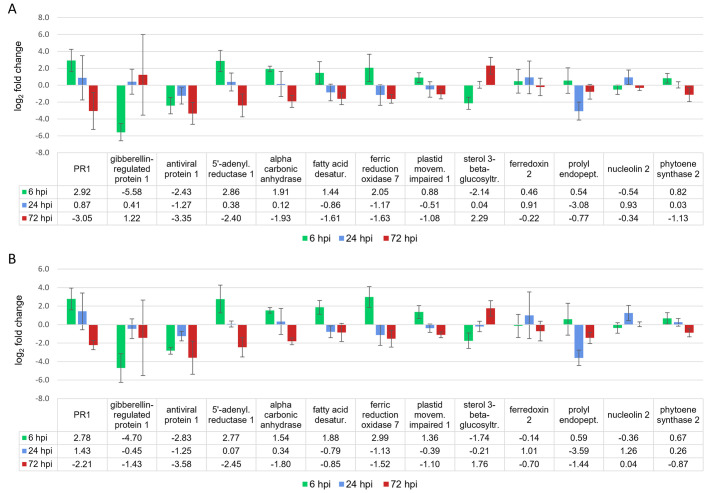
Time course of thirteen selected sugar beet genes deregulated after aphid-mediated BYV inoculation shown for RT-qPCR validation (A) and biological repetition (B) samples. Standard deviation is based on n = 4.

Considering the time course in RT-qPCR and biological repetition samples, the general expression patterns of the selected genes were very similar for both sample types. PR-1, and the majority of chloroplast-related genes were significantly up-regulated at 6 hpi and significantly down-regulated at 72 hpi in at least one of the two sample types, with the exception of phytoene synthase 2. The gibberellin-regulated protein 1 and the sterol 3-beta-glucosyltransferase showed significant down-regulation at 6 hpi, but only the latter gene was significantly up-regulated at 72 hpi. Prolyl endopeptidase was significantly down-regulated at 24 hpi and 72 hpi (only in the repetition sample). Ferredoxin 2 and nucleolin 2 did not show significant expression. The antiviral protein 1 was significantly down-regulated at 6 and 72 hpi. (Fig 9).

## 4. Discussion

Our study is the first to show transcriptional plant responses following natural aphid-mediated transmission of the ssRNA virus BYV in sugar beet focusing on the initial phase of infection. It makes a significant contribution to the identification of potential key genes in the compatible sugar beet–BYV interaction, especially in the initiation and establishment of virus-induced disease processes.

### 4.1 Putative proviral factors

Components of cell wall metabolism can affect the systemic spread of the virus [31]. We identified modifications of cellulose, hemicellulose, pectin, lignin and cutin/suberin as well as cell wall protein (expansin) deregulation. All these identified genes were significantly up-regulated, however, exclusively in our 24 hpi dataset.

In a cassava transcriptome study investigating responses to infection with South African cassava mosaic virus (SACMV, *Geminiviridae*), host genes encoding cell-wall polysaccharides, and expression of plasmodesmata-associated genes were up-regulated. The up-regulation was linked to the increase of SACMV titers between earlier and later sampling time points, suggesting a role in SACMV movement [32]. In our dataset, at 6 hpi and 72 hpi, the majority of cell wall-associated DEGs were down-regulated. In the compatible tomato spotted wilt virus-tomato interaction, cell wall modification enzymes such as xyloglucane, endotransglycosylase/hydrolases and pectinesterase were also down-regulated [33]. Furthermore, in rice plants infected with rice dwarf virus (RDV, *Sedoreoviridae*), the most affected pathway was the cell wall-related pathway. Corresponding genes were significantly suppressed, and it was suggested that this might reflect the stunted growth of diseased plants [34]. In our data, the later time point (72 hpi) also showed down-regulation of a polygalacturonase (bv6_148680_pnhi.t1; -2.714) and a regulatory protein of the cellulose-hemicellulose network COB (bv2_028020_grdy.t1; -1.897). The deregulation of cell wall-related genes is therefore a possible explanation for the generally more compact growth and smaller leaf area, and indicates that these genes might be involved in viral movement.

Furthermore, we identified transcription factors (TFs) of different classes that played a role in the very early phase of infection. TFs have a substantial impact on controlling cellular processes such as responses to biotic and abiotic stresses, development, cell differentiation, metabolism and defense [35]. In particular, the MYB class-R2R3 subgroup-18 transcription factor GAMYB, which has a function in hormone response and gibberellic acid (GA) signal transduction [35], showed high differential expression (bv_000790_ezhe.t1; 4.146).

A chaperone heat-shock (Hsp70) protein (bv7_174160_nend.t2; -2.302) and class-C-II and class-M-I small heat-shock responsive proteins (bv2_025750_azsu.t1, bv9_205180_adke.t1, bv2_034220_scas.t1, bv5_123200_druz.t1; -2.121 to -5.069) were down-regulated. Controversially to our study, heat-shock proteins have been found to be induced, e.g. by the negative-sense ssRNA rice stripe virus [36], the positive-sense ssRNA viruses turnip mosaic virus (TuMV, *Potyviridae*) and turnip crinkle virus (TCV, *Tombusviridae*) [37], and the DNA virus tomato yellow leaf curl virus (TYLCV, *Geminiviridae*) [38, 39]. These findings suggest proviral activity, since viral recruitment of cellular host chaperones to membrane bound sites seems to be essential for virus replication and cell-to-cell movement, making such chaperones and heat-shock proteins potential targets for antiviral defense [40]. However, HSP70 genes were down-regulated upon infection of *Vitis* with grapevine leafroll-associated virus 3 (GLRaV-3, *Closteroviridae*) [41]. Like BYV, GLRaV-3 is a closterovirus, which is phloem-restricted and semi-persistently transmitted by phloem-sap sucking mealybugs [41]. HSP70s can be localized in the ER and cytosol or the mitochondria and chloroplasts. It was shown that cytosolic and chloroplastic HSP70s were predominantly repressed following biotic stressors [42]. Targeting of different HSP70s in different cell organelles might explain the down-regulation of these host genes representing another strategy used by the closteroviruses to evade host stress responses [42].

The solute transporter UmamiT was highly up-regulated at 72 hpi (bv3_049340_cfxz.t1, 5.16). UmamiT was investigated in Arabidopsis transgenic over-expression lines, which showed stunted growth [43]. Further studies of such over-expressor lines, showed altered physiological characteristics such as a decline in biomass and seed yield. Resistance, in this case against the fungus *Phytophthora parasitica*, was shown for mutants defective in UmamiT [43] so that screening for natural mutations might be an option for implementing resistant phenotypes.

It is assumed that some viruses circumvent the plant silencing (RNAi) mechanism by physically escaping and replicating in vesicles or chloroplasts [44]. The trafficking of vesicles is facilitated by a superfamily of proteins known as SNAREs (soluble N-ethylmaleimide-sensitive-factor attachment protein receptors). SNAREs are possibly involved in turnip mosaic virus (TuMV) infection [45, 46] as vesicle are attached with the outer membrane of chloroplasts. Viral structures co-localize with vesicles on the chloroplast membrane [45]. The SNARE gene Syp71 mediates the formation of bridge-like structures between two chloroplasts, probably facilitating the trafficking of virus particles [44]. Knockdown of Syp71 using a tobacco rattle virus (TRV, *Virgaviridae*) -based virus-induced gene silencing vector showed that Syp71 is essential for TuMV infection. In Syp71 down-regulated plant cells, virus accumulation was significantly reduced [45]. In our data, we identified a vesicle trafficking regulation protein belonging to the Syp1-group (bv_000340_yrnu.t1; 5.184) being highly up-regulated that might play a role in BYV replication and trafficking.

### 4.2 Phytohormone deregulation and antiviral proteins

There is emerging evidence that viruses manipulate plant hormone responses to deactivate defense responses and reprogram cells to enhance viral replication and spread [30]. This involves hormonal cross-talk during viral pathogenesis [47]. Our data suggests cross-talk between the different phytohormone pathways in the early stages of BYV infection. We found that SA- and ET-related genes were not deregulated. The ABA pathway appeared to be of greater importance in the very early stages of infection. Three genes showed up-regulation at 6 or 24 hpi, while JA-related genes were down-regulated at 72 hpi. The deregulation appears to be characteristic for viral infections. It is already known that virus infections stimulate the ABA synthesis and disturb other defense-related hormonal pathways including SA, ET, or JA synthesis [47]. CKs also play an essential role in the growth processes of plants, as increased CK levels enhance the resistance against viruses [47]. However, only few reports exist describing the response of CK pathways to virus infection [48]. In our transcriptome dataset, the majority of CK-related genes were significantly down-regulated at 6 and especially at 72 hpi. The interaction between GAs and Aux upon viral infection is unclear [48], however, our data suggest that both pathways are induced having antagonistic effects.

Furthermore, we identified a putative antiviral protein alpha (bv9_202460_padn.t1; -2.952) that was significantly down-regulated at 6 and 72 hpi. It was also down-regulated at 24 hpi, but not significantly between the four sample repetitions. Antiviral proteins are defense-related proteins belonging to the so-called ribosome-inactivating proteins (RIPs) and are known to inhibit protein synthesis. From a structural point of view, they have been classified into two types: type 1 RIPs are for example saporin, pokeweed antiviral protein (PAP) [49–51], PD-L1 and Beetin 27 (BE27), the latter can be found in sugar beet (bv9_202430), and type 2 RIPs (e.g. ricin, abrin, mistletoe lectin) [52]. The down-regulation of antiviral protein alpha might indicate the susceptibility of the genotype tested.

### 4.3 Chloroplasts- and photosynthesis-related genes

Although the development of viral symptoms can have multiple causes, the disturbance of normal chloroplast function has been suggested to cause typical photosynthesis-related symptoms, such as mosaic and chlorosis [53]. It seems plausible that deregulation of chlorophyll biosynthesis genes plays a role in the induction of yellowing symptoms, since yellowing increases the attractiveness for insect vector colonization [54] and thus transmission and spread. It is already well known that chloroplast- and photosynthesis-related genes or proteins, respectively, are common targets assisting in viral propagation [55]. It is interesting to mention that plant viruses can also exploit host RNA silencing machinery to manipulate chlorophyll-related genes at the post-transcription level [55]. In tobacco, the chlorophyll biosynthesis gene *CHLI* was found to be specifically down-regulated by cucumber mosaic virus (CMV, *Bromoviridae*) + Y-Sat infection. As a result, the virus is able to induce the typical yellowing symptoms [56].

For BYV as a virus that causes leaf yellowing or chlorosis, we were particularly interested, if plant genes involved in the expression of symptoms caused by the viral infection can already be identified in the early infection phase.

In our data set, we found that 6% of all deregulated genes were related to photosynthesis or chloroplasts. All these DEGs mainly had in common that they were down-regulated at the latest of the early infection time points investigated. Thus, it can be assumed that chloroplast-related genes are deregulated by BYV early in the infection process and on locally infected tissue. Screening the available early transcriptomic studies, reveals the importance of chlorophyll- and chloroplast-related genes.

Kundu et al. (2015) identified altered expression of photsynthesis-related genes in black gram (*Vigna mungo*) 3-48 hours after infection with mungbean yellow mosaic india virus (MYMIV, *Geminiviridae*), transmitted by the viruliferous whitefly *Bemisia tabaci*. These genes were mainly repressed, reflecting the lower photosynthetic competence accompanying susceptible phenotypes showing severe chlorosis [57], which is consistent with our observations.

Cho et al. (2015) studied the transcriptional changes in rice plants triggered by rice stripe virus (RSV, *Phenuiviridae*) transmitted by planthoppers. They showed that many chloroplast-related genes were down-regulated quite soon (3 dpi) after RSV infection, even though the infected rice seedlings did not show any disease symptoms, suggesting that down-regulation of chloroplast-related genes reduces photosynthesis, which in turn leads to chlorosis on infected leaves [58].

Another study, though using mechanical inoculation of leaves, dealt with potato virus Y (PVY, *Potyviridae*) infection in potato and discovered the biological process GO term “Photosynthesis, light harvesting” to be the most overrepresented term in potato leaves inoculated with PVY already at 4 hpi and the majority of photosynthesis-related genes were down-regulated at 10 and 12 hpi [59]. Thus, our study and others show the importance of this pathway for the plant-virus interaction for different virus families.

Our analysis clearly demonstrates that gene deregulation processes in the compatible sugar beet-BYV interaction are already activated at a very early stage of infection following natural vector inoculation. In the abundance of DEGs, it is a difficult task to subsequently identify possible susceptibility factors. Regulatory processes are complex and can also link several factors or genes. From the list of DEGs, it is possible to carefully select which genes might be interesting for a more detailed characterization. As the BIN categories are generally very broadly covered, the commonly regulated genes may offer a narrower selection. Selected genes can then be further investigated. Possible future experiments can be virus-induced gene silencing using VIGS vectors to see whether the silencing of certain genes, which might be essential factors for virus replication and spread in the plant, can trigger tolerance or resistance effects. It is also possible to delete or specifically modify such factors using CRISPR/Cas and test the mutant plants for susceptibility or resistance. Using this approach, we have recently identified resistance against BChV, another pathogen of the virus yellows complex, attributed to a deletion in the eukaryotic translation initiation factor eIF(iso)4E that negatively interferes with the viral protein VPg (viral genome-linked protein), which mediates translation of viral proteins [60].

Such identification of susceptibility factors can support sugar beet breeders to develop BYV-resistant varieties for the market and thereby eliminating a threat to sugar beet cultivation, as no varieties with effective resistance are currently available.

## Supporting information

S1 Fig Detection of the housekeeping gene glyceraldehyde-3-phosphate-dehydrogenase (149 bp) and BYV (823 bp) in RNA/cDNA transcriptomic back up samples. B5-B8 = 6 hpi (hours post inoculation) mock-inoculated samples, C5-C8 = 6 hpi BYV-inoculated; D5-D8 = 24 hpi mock-inoculated, E5-E8 = 24 hpi BYV-inoculated; F5-F8 = 72 hpi mock-inoculated, G9-G12 = 72 hpi BYV-inoculated samples. Negative control (-), positive control (+).
(TIF)

S2 Fig Detection of the housekeeping gene glyceraldehyde-3-phosphate-dehydrogenase (149 bp) and BYV (823 bp) in RNA/cDNA biological repetition samples. B5-B8 = 6 hpi (hours post inoculation) mock-inoculated samples, C5-C8 = 6 hpi BYV-inoculated; D5-D8 = 24 hpi mock-inoculated, E5-E8 = 24 hpi BYV-inoculated; F5-F8 = 72 hpi mock-inoculated, G9-G12 = 72 hpi BYV-inoculated samples. Negative control (-), positive control (+).
(TIF)

S1 Table List of primers that were used for gene expression validation analysis by RT-qPCR. (XLSX)

S2 Table Mapping results. (XLSX)

S3 Table Significantly enriched BIN categories of differentially expressed genes (DEGs; up- ↑ or down- ↓ regulated) in BYV-inoculated plants 6, 24 and 72 hours post inoculation (hpi). (DOCX)

S4 Table Significantly enriched BIN categories of common differentially expressed genes (DEGs; up- ↑ or down- ↓ regulated) in BYV-inoculated plants 6, 24 and 72 hours post inoculation (hpi). (DOCX)

S5 Table Chloroplast-related genes that were solely or commonly identified 6, 24 and 72 hours after BYV inoculation. (DOCX)

S6 Table List of DEGs with higher log2 fold change than -5 or 5. (XLSX)

S7 Table List of genes for RT-qPCR gene expression validation and significance levels in RT-qPCR validation and biological repetition samples. (XLSX)

## References

[1] Hossain R, Menzel W, Lachmann C, Varrelmann M. New insights into virus yellows distribution in Europe and effects of beet yellows virus, beet mild yellowing virus, and beet chlorosis virus on sugar beet yield following field inoculation. Plant Pathology 2021; 70(3):584–93.

[2] Smith HG, Hallsworth PB. The effects of yellowing viruses on yield of sugar beet in field trials, 1985 and 1987. Annals of Applied Biology 1990; 116(3):503–11.

[3] d Koeijer KJ, van der Werf W. Effects of beet yellows virus and beet mild yellowing virus on leaf area dynamics of sugar beet (Beta vulgaris L.). Field Crops Research 1999; 61(2):163–77.

[4] Clover GRG, Azam-Ali SN, Jaggard KW, Smith HG. The effects of beet yellows virus on the growth and physiology of sugar beet (Beta vulgaris). Plant Pathology 1999; 48(1).

[5] Esau K, Cronshaw J, Hoefert LL. Relation of beet yellows virus to the phloem and to movement in the sieve tube. J Cell Biol 1967; 32(1):71–87. doi: 10.1083/jcb.32.1.71 10976202 PMC2107099

[6] Bennett CW. Sugar beet yellows disease in the United States. US Department of Agriculture; 1960. (vol 1218).

[7] Duffus JE. The Yellowing Virus Diseases of Beet. Advances in Virus Research 1973; 18:347–86.

[8] Agranovsky AA, Koonin EV, Boyko VP, Maiss E, Frötschl R, Lunina NA et al. Beet yellows closterovirus: complete genome structure and identification of a leader papain-like thiol protease. Virology 1994; 198(1):311–24. doi: 10.1006/viro.1994.1034 8259666

[9] Limburg DD, Mauk PA, Godfrey LD. Characteristics of Beet Yellows Closterovirus Transmission to Sugar Beets by Aphis fabae. Phytopathology 1997; 87(7):766–71. doi: 10.1094/PHYTO.1997.87.7.766 18945100

[10] Agranovsky AA, Folimonov AS, Folimonova SY, Morozov SY, Schiemann J, Lesemann D et al. Beet yellows closterovirus HSP70-like protein mediates the cell-to-cell movement of a potexvirus transport-deficient mutant and a hordeivirus-based chimeric virus. J Gen Virol 1998; 79 (Pt 4):889–95. doi: 10.1099/0022-1317-79-4-889 9568985

[11] Karasev AV, Kashina AS, Gelfand VI, Dolja VV. HSP70-related 65 kDa protein of beet yellows closterovirus is a microtubule-binding protein. FEBS Letters 1992; 304(1):12–4. doi: 10.1016/0014-5793(92)80578-5 1618294

[12] Agranovsky AA, Lesemann DE, Maiss E, Hull R, Atabekov JG. "Rattlesnake" structure of a filamentous plant RNA virus built of two capsid proteins. Proceedings of the National Academy of Sciences 1995; 92(7):2470–3. doi: 10.1073/pnas.92.7.2470 7708667 PMC42239

[13] Chiba M, Reed JC, Prokhnevsky AI, Chapman EJ, Mawassi M, Koonin EV et al. Diverse suppressors of RNA silencing enhance agroinfection by a viral replicon. Virology 2006; 346(1):7–14. doi: 10.1016/j.virol.2005.09.068 16300814

[14] Agranovsky AA. Closteroviruses: Molecular Biology, Evolution and Interactions with Cells. In: Gaur RK, Petrov NM, Patil BL, Stoyanova MI, editors. Plant Viruses: Evolution and Management. Singapore: Springer Singapore; 2016. p. 231–52.

[15] Haniotakis GE, Lange WH. Beet Yellows Virus Resistance in Sugar Beets: Mechanism of Resistance. J Econ Entomol 1974; 67(1):25–8.

[16] Grimmer MK, Bean KMR, Qi A, Stevens M, Asher MJC. The action of three Beet yellows virus resistance QTLs depends on alleles at a novel genetic locus that controls symptom development. Plant Breeding 2008; 127(4):391–7.

[17] Stevanato P, Chiodi C, Biancardi E, Pavli O. Broccanello C., Skaracis G, Concheri G. Sustainability of the Sugar Beet Crop. Sugar Tech 2019; 21(5).

[18] Garcia-Ruiz H. Susceptibility Genes to Plant Viruses. Viruses 2018; 10(9):484. doi: 10.3390/v10090484 30201857 PMC6164914

[19] Dohm JC, Minoche AE, Holtgräwe D, Capella-Gutiérrez S, Zakrzewski F, Tafer H et al. The genome of the recently domesticated crop plant sugar beet (Beta vulgaris). Nature 2014; 505(7484):546–9. doi: 10.1038/nature12817 24352233

[20] Zanardo LG, de Souza GB, Alves MS. Transcriptomics of plant–virus interactions: a review. Theor. Exp. Plant Physiol. 2019; 31(1):103–25. 10.1007/s40626-019-00142-0 PMC6660014 31355128

[21] Ward JA, Ponnala L, Weber CA. Strategies for transcriptome analysis in nonmodel plants. American Journal of Botany 2012; 99(2):267–76. doi: 10.3732/ajb.1100334 22301897

[22] Schmidlin L, de Bruyne E, Weyens G, Lefebvre M, Gilmer D. Identification of differentially expressed root genes upon rhizomania disease. Mol Plant Pathol 2008; 9(6):741–51. doi: 10.1111/j.1364-3703.2008.00498.x 19019003 PMC6640463

[23] Fernando Gil J, Wibberg D, Eini O, Savenkov EI, Varrelmann M, Liebe S. Comparative Transcriptome Analysis Provides Molecular Insights into the Interaction of Beet necrotic yellow vein virus and Beet soil-borne mosaic virus with Their Host Sugar Beet. Viruses 2020; 12(1). doi: 10.3390/v12010076 31936258 PMC7019549

[24] Yousefiara M, Malboobi MA, Bagheri A, Moshtaghi N. Comparative Transcriptome Analyses of a Transgenic Sugar Beet Resistant to Beet Necrotic Yellow Vein Virus. Journal of Cell and Molecular Research 2022; 14(1):44–53.

[25] Decroës A, Mahillon M, Genard M, Lienard C, Lima-Mendez G, Gilmer D. et al. Rhizomania: Hide and Seek of Polymyxa betae and the Beet Necrotic Yellow Vein Virus with Beta vulgaris. Mol Plant Microbe Interact 2022; 35(11):989–1005. doi: 10.1094/MPMI-03-22-0063-R 35816413

[26] Clark MF, Adams AN. Characteristics of the microplate method of enzyme-linked immunosorbent assay for the detection of plant viruses. Journal of General Virology 1977; 34(3):475–83. doi: 10.1099/0022-1317-34-3-475 323416

[27] Thimm O, Bläsing O, Gibon Y, Nagel A, Meyer S, Krüger P et al. mapman: a user-driven tool to display genomics data sets onto diagrams of metabolic pathways and other biological processes. The Plant Journal 2004; 37(6):914–39. doi: 10.1111/j.1365-313x.2004.02016.x 14996223

[28] Klie S, Nikoloski Z. The Choice between MapMan and Gene Ontology for Automated Gene Function Prediction in Plant Science. Front Genet 2012; 3:115. doi: 10.3389/fgene.2012.00115 22754563 PMC3384976

[29] Livak KJ, Schmittgen TD. Analysis of Relative Gene Expression Data Using Real-Time Quantitative PCR and the 2−ΔΔCT Method. Methods 2001; 25(4):402–8. 11846609 10.1006/meth.2001.1262

[30] Collum TD, Culver JN. The impact of phytohormones on virus infection and disease. Current Opinion in Virology 2016; 17:25–31. doi: 10.1016/j.coviro.2015.11.003 26656395

[31] Kozieł E, Otulak-Kozieł K, Bujarski JJ. Plant Cell Wall as a Key Player During Resistant and Susceptible Plant-Virus Interactions. Front Microbiol 2021; 12:656809. doi: 10.3389/fmicb.2021.656809 33776985 PMC7994255

[32] Allie F, Pierce EJ, Okoniewski MJ, Rey C. Transcriptional analysis of South African cassava mosaic virus-infected susceptible and tolerant landraces of cassava highlights differences in resistance, basal defense and cell wall associated genes during infection. BMC Genomics 2014; 15(1):1006. doi: 10.1186/1471-2164-15-1006 25412561 PMC4253015

[33] Nachappa P, Challacombe J, Margolies DC, Nechols JR, Whitfield AE, Rotenberg D. Tomato Spotted Wilt Virus Benefits Its Thrips Vector by Modulating Metabolic and Plant Defense Pathways in Tomato. Front Plant Sci 2020; 11:575564. doi: 10.3389/fpls.2020.575564 33424878 PMC7793759

[34] Shimizu T, Satoh K, Kikuchi S, Omura T. The repression of cell wall- and plastid-related genes and the induction of defense-related genes in rice plants infected with Rice dwarf virus. Mol Plant Microbe Interact 2007; 20(3):247–54. doi: 10.1094/MPMI-20-3-0247 17378427

[35] Ambawat S, Sharma P, Yadav NR, Yadav RC. MYB transcription factor genes as regulators for plant responses: an overview. Physiol Mol Biol Plants 2013; 19(3):307–21. doi: 10.1007/s12298-013-0179-1 24431500 PMC3715649

[36] Jiang S, Lu Y, Li K, Lin L, Zheng H, Yan F et al. Heat shock protein 70 is necessary for Rice stripe virus infection in plants. Mol Plant Pathol 2014; 15(9):907–17. doi: 10.1111/mpp.12153 24823923 PMC6638618

[37] Aparicio F, Thomas CL, Lederer C, Niu Y, Wang D, Maule AJ. Virus induction of heat shock protein 70 reflects a general response to protein accumulation in the plant cytosol. Plant Physiol 2005; 138(1):529–36. doi: 10.1104/pp.104.058958 15805473 PMC1104204

[38] Gorovits R, Moshe A, Ghanim M, Czosnek H. Recruitment of the host plant heat shock protein 70 by Tomato yellow leaf curl virus coat protein is required for virus infection. PLOS ONE 2013; 8(7):e70280. doi: 10.1371/journal.pone.0070280 23894631 PMC3720902

[39] Gorovits R, Czosnek H. The Involvement of Heat Shock Proteins in the Establishment of Tomato Yellow Leaf Curl Virus Infection. Front Plant Sci 2017; 8:355. doi: 10.3389/fpls.2017.00355 28360921 PMC5352662

[40] Verchot J. Cellular chaperones and folding enzymes are vital contributors to membrane bound replication and movement complexes during plant RNA virus infection. Front Plant Sci 2012; 3:275. doi: 10.3389/fpls.2012.00275 23230447 PMC3515963

[41] Prator CA, Chooi KM, Jones D, Davy MW, MacDiarmid RM, Almeida RPP. Comparison of two different host plant genera responding to grapevine leafroll-associated virus 3 infection. Sci Rep 2020; 10(1):8505. doi: 10.1038/s41598-020-64972-8 32444786 PMC7244584

[42] Berka M, Kopecká R, Berková V, Brzobohatý B, Černý M. Regulation of heat shock proteins 70 and their role in plant immunity. J Exp Bot 2022; 73(7):1894–909. doi: 10.1093/jxb/erab549 35022724 PMC8982422

[43] Besnard J, Sonawala U, Maharjan B, Collakova E, Finlayson SA, Pilot G et al. Increased Expression of UMAMIT Amino Acid Transporters Results in Activation of Salicylic Acid Dependent Stress Response. Front Plant Sci 2020; 11:606386. doi: 10.3389/fpls.2020.606386 33574824 PMC7870477

[44] Bhattacharyya D, Chakraborty S. Chloroplast: the Trojan horse in plant-virus interaction. Mol Plant Pathol 2018; 19(2):504–18. doi: 10.1111/mpp.12533 28056496 PMC6638057

[45] Wei T, Zhang C, Hou X, Sanfaçon H, Wang A. The SNARE protein Syp71 is essential for turnip mosaic virus infection by mediating fusion of virus-induced vesicles with chloroplasts. PLoS Pathog 2013; 9(5):e1003378. doi: 10.1371/journal.ppat.1003378 23696741 PMC3656112

[46] Cabanillas DG, Jiang J, Movahed N, Germain H, Yamaji Y, Zheng H et al. Turnip Mosaic Virus Uses the SNARE Protein VTI11 in an Unconventional Route for Replication Vesicle Trafficking. Plant Cell 2018; 30(10):2594–615. doi: 10.1105/tpc.18.00281 30150314 PMC6241277

[47] Mishra J, Srivastava R, Trivedi PK, Verma PC. Effect of virus infection on the secondary metabolite production and phytohormone biosynthesis in plants. 3 Biotech 2020; 10(12):547. doi: 10.1007/s13205-020-02541-6 33269181 PMC7683645

[48] Zhao S, Li Y. Current understanding of the interplays between host hormones and plant viral infections. PLOS Pathogens 2021; 17(2):e1009242. doi: 10.1371/journal.ppat.1009242 33630970 PMC7906326

[49] Di R, Tumer NE. Pokeweed antiviral protein: its cytotoxicity mechanism and applications in plant disease resistance. Toxins (Basel) 2015; 7(3):755–72. doi: 10.3390/toxins7030755 25756953 PMC4379523

[50] Zhu F, Yuan S, Zhang Z-W, Qian K, Feng J-G, Yang Y-Z. Pokeweed antiviral protein (PAP) increases plant systemic resistance to Tobacco mosaic virus infection in Nicotiana benthamiana. Eur J Plant Pathol 2016; 146(3):541–9.

[51] Neller KCM, Diaz CA, Platts AE, Hudak KA. De novo Assembly of the Pokeweed Genome Provides Insight Into Pokeweed Antiviral Protein (PAP) Gene Expression. Front Plant Sci 2019; 10:1002. doi: 10.3389/fpls.2019.01002 31447869 PMC6691146

[52] Iglesias R, Citores L, Di Maro A, Ferreras JM. Biological activities of the antiviral protein BE27 from sugar beet (Beta vulgaris L.). Planta 2015; 241(2):421–33. doi: 10.1007/s00425-014-2191-2 25326773

[53] Rahoutei J, García‐Luque I, Barón M. Inhibition of photosynthesis by viral infection: Effect on PSII structure and function. Physiol Plant 2000; 110(2):286–92.

[54] Chesnais Q, Sun P, Mauck KE. Advanced infections by cucurbit yellow stunting disorder virus encourage whitefly vector colonization while discouraging non-vector aphid competitors. J Pest Sci 2022; 95(1):231–47.

[55] Zhao J, Zhang X, Hong Y, Liu Y. Chloroplast in Plant-Virus Interaction. Front Microbiol 2016; 7:1565. doi: 10.3389/fmicb.2016.01565 27757106 PMC5047884

[56] Smith NA, Eamens AL, Wang M-B. Viral small interfering RNAs target host genes to mediate disease symptoms in plants. PLoS Pathog 2011; 7(5):e1002022. doi: 10.1371/journal.ppat.1002022 21573142 PMC3088724

[57] Kundu A, Patel A, Paul S, Pal A. Transcript dynamics at early stages of molecular interactions of MYMIV with resistant and susceptible genotypes of the leguminous host, Vigna mungo. PLOS ONE 2015; 10(4):e0124687. doi: 10.1371/journal.pone.0124687 25884711 PMC4401676

[58] Cho WK, Lian S, Kim S-M, Seo BJ, Jung JK, Kim K-H. Time-Course RNA-Seq Analysis Reveals Transcriptional Changes in Rice Plants Triggered by Rice stripe virus Infection. PLOS ONE 2015; 10(8):e0136736. doi: 10.1371/journal.pone.0136736 26305329 PMC4549299

[59] Goyer A, Hamlin L, Crosslin JM, Buchanan A, Chang JH. RNA-Seq analysis of resistant and susceptible potato varieties during the early stages of potato virus Y infection. BMC Genomics 2015; 16(1):472. doi: 10.1186/s12864-015-1666-2 26091899 PMC4475319

[60] Rollwage L, van Houtte H, Hossain R, Wynant N, Willems G, Varrelmann M. Recessive resistance against beet chlorosis virus is conferred by the eukaryotic translation initiation factor (iso)4E in Beta vulgaris. Plant Biotechnology Journal 2024. doi: 10.1111/pbi.14333 38488845 PMC11258979

