# Supplementary figures and images for: Aphid-mediated beet yellows virus transmission initiates proviral gene deregulation in sugar beet at early stages of infection
Source: PLoS One. 2024 Oct 1;19(10):e0311368. doi: 10.1371/journal.pone.0311368 (PMC11444407; doi:10.1371/journal.pone.0311368)

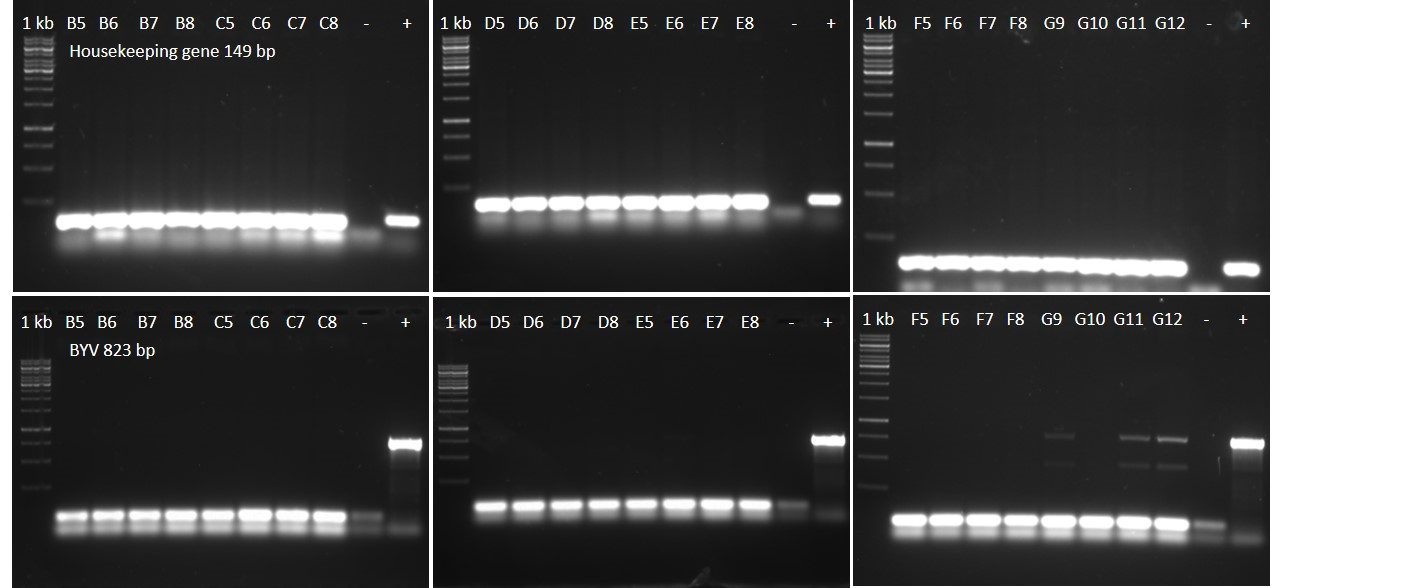

Supplement: S1 Fig — B5-B8 = 6 hpi (hours post inoculation) mock-inoculated samples, C5-C8 = 6 hpi BYV-inoculated; D5-D8 = 24 hpi mock-inoculated, E5-E8 = 24 hpi BYV-inoculated; F5-F8 = 72 hpi mock-inoculated, G9-G12 = 72 hpi BYV-inoculated samples. Negative control (-), positive control (+). (TIF) [file pone.0311368.s001.tif]

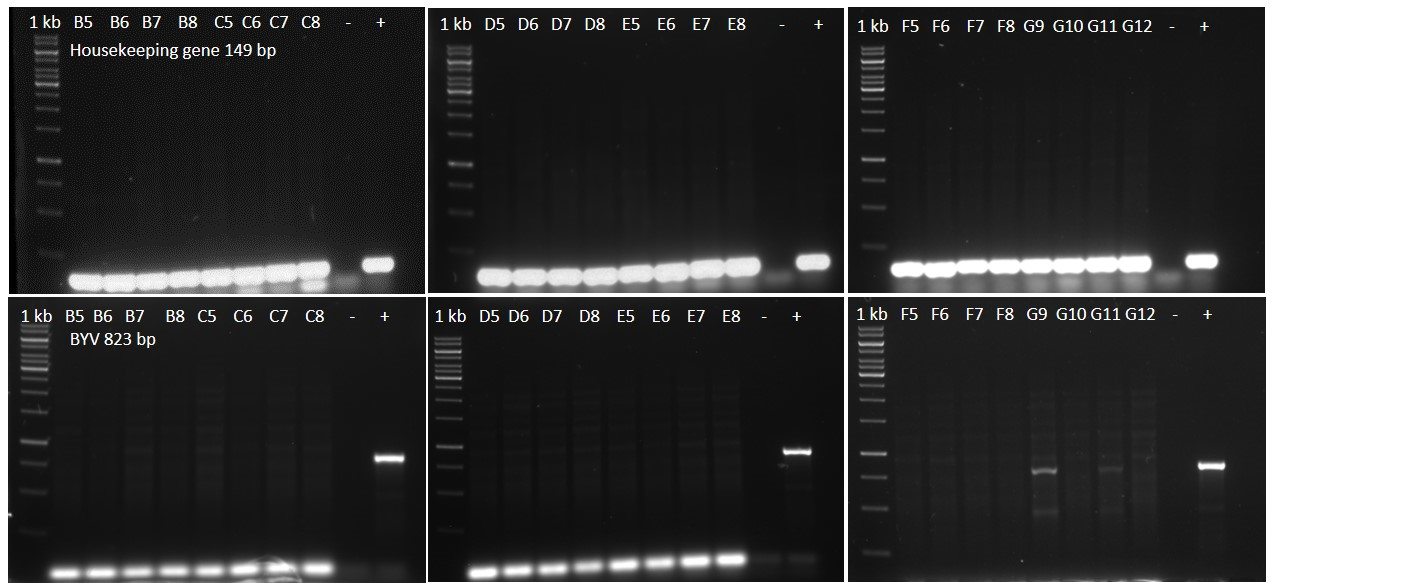

Supplement: S2 Fig — B5-B8 = 6 hpi (hours post inoculation) mock-inoculated samples, C5-C8 = 6 hpi BYV-inoculated; D5-D8 = 24 hpi mock-inoculated, E5-E8 = 24 hpi BYV-inoculated; F5-F8 = 72 hpi mock-inoculated, G9-G12 = 72 hpi BYV-inoculated samples. Negative control (-), positive control (+). (TIF) [file pone.0311368.s002.tif]
