# Supplementary material for: Aphid-mediated beet yellows virus transmission initiates proviral gene deregulation in sugar beet at early stages of infection
Source: PLoS One. 2024 Oct 1;19(10):e0311368. doi: 10.1371/journal.pone.0311368 (PMC11444407; doi:10.1371/journal.pone.0311368)
Supplement: S3 Table — (DOCX) [file pone.0311368.s005.docx]

| Suppl. Tab. S3: Significantly enriched BIN categories of differentially expressed genes (DEGs; up- ↑ or down- ↓ regulated) in BYV-inoculated plants 6, 24 and 72 hours post inoculation (hpi). | | | | | | | | | | |
| --- | --- | --- | --- | --- | --- | --- | --- | --- | --- | --- |
| BIN | **Category** | **No. of DEGs** | | | | | | | | |
|  |  | **6 hpi** | | | **24 hpi** | | | **72 hpi** | | |
|  |  | All | ↑ | ↓ | All | ↑ | ↓ | All | ↑ | ↓ |
| 1 | Photosynthesis | 3 | 3 | 0 | 1 | 1 | 0 | 1 | 0 | 1 |
| 2 | Cellular respiration | 1 | 1 | 0 | 0 | 0 | 0 | 0 | 0 | 0 |
| 3 | Carbohydrate metabolism | 7 | 2 | 5 | 4 | 4 | 0 | 2 | 0 | 2 |
| 4 | Amino acid metabolism | 1 | 1 | 0 | 1 | 1 | 0 | 2 | 0 | 2 |
| 5 | Lipid metabolism | 6 | 3 | 3 | 2 | 2 | 0 | 3 | 0 | 3 |
| 6 | Nucleotide metabolism | 1 | 0 | 1 | 0 | 0 | 0 | 1 | 0 | 1 |
| 7 | Coenzyme metabolism | 2 | 2 | 0 | 0 | 0 | 0 | 2 | 0 | 2 |
| 8 | Polyamine metabolism | 1 | 1 | 0 | 0 | 0 | 0 | 3 | 1 | 2 |
| 9 | Secondary metabolite | 0 | 0 | 0 | 1 | 1 | 0 | 3 | 0 | 3 |
| 10 | Redox homeostasis | 0 | 0 | 0 | 3 | 3 | 0 | 0 | 0 | 0 |
| 11 | Phytohormon action | 10 | 5 | 5 | 5 | 5 | 0 | 11 | 4 | 7 |
| 12 | Chromatin organization | 2 | 2 | 0 | 3 | 3 | 0 | 2 | 1 | 1 |
| 13 | Cell division | 0 | 0 | 0 | 6 | 6 | 0 | 3 | 2 | 1 |
| 14 | DNA damage response | 2 | 2 | 0 | 0 | 0 | 0 | 0 | 0 | 0 |
| 15 | RNA biosynthesis | 13 | 8 | 5 | 8 | 7 | 1 | 8 | 4 | 4 |
| 16 | RNA processing | 2 | 2 | 0 | 3 | 3 | 0 | 5 | 1 | 4 |
| 17 | Protein biosynthesis | 1 | 1 | 0 | 0 | 0 | 0 | 0 | 0 | 0 |
| 18 | Protein modification | 4 | 2 | 2 | 7 | 7 | 0 | 6 | 3 | 3 |
| 19 | Protein homeostasis | 6 | 2 | 4 | 7 | 6 | 1 | 10 | 2 | 8 |
| 20 | Cytoskeleton organization | 3 | 2 | 1 | 5 | 5 | 0 | 3 | 0 | 3 |
| 21 | Cell wall organization | 7 | 1 | 6 | 18 | 18 | 0 | 3 | 0 | 3 |
| 22 | Vesicle trafficking | 0 | 0 | 0 | 1 | 1 | 0 | 0 | 0 | 0 |
| 23 | Protein translocation | 0 | 0 | 0 | 0 | 0 | 0 | 0 | 0 | 0 |
| 24 | Solute transport | 11 | 6 | 5 | 4 | 3 | 1 | 10 | 3 | 7 |
| 25 | Nutrient uptake | 3 | 2 | 1 | 0 | 0 | 0 | 0 | 0 | 0 |
| 26 | External stimuli response | 4 | 2 | 2 | 2 | 2 | 0 | 3 | 1 | 2 |
| 27 | Multi-process regulation | 5 | 3 | 2 | 4 | 3 | 1 | 2 | 1 | 1 |
| 28 | Plant reproduction | 1 | 0 | 1 | 0 | 0 | 0 | 0 | 0 | 0 |
| 30 | clade-specific metabolism | 0 | 0 | 0 | 0 | 0 | 0 | 0 | 0 | 0 |
| 35 | not assigned | 88 | 52 | 36 | 76 | 70 | 6 | 66 | 23 | 43 |
| 50 | enzyme classification | 16 | 9 | 7 | 18 | 17 | 1 | 4 | 1 | 3 |
|  | **Total** | 200 | 114 | 86 | 179 | 168 | 11 | 153 | 47 | 106 |
