# Supplementary material for: Aphid-mediated beet yellows virus transmission initiates proviral gene deregulation in sugar beet at early stages of infection
Source: PLoS One. 2024 Oct 1;19(10):e0311368. doi: 10.1371/journal.pone.0311368 (PMC11444407; doi:10.1371/journal.pone.0311368)
Supplement: S4 Table — (DOCX) [file pone.0311368.s006.docx]

| Suppl. Tab. S4: Significantly enriched BIN categories of common differentially expressed genes (DEGs; up- ↑ or down- ↓ regulated) in BYV-inoculated plants 6, 24 and 72 hours post inoculation (hpi). | | | | | | | | | | |
| --- | --- | --- | --- | --- | --- | --- | --- | --- | --- | --- |
| BIN | **Category** | **No. of DEGs** | | | | | | | | |
|  |  | **6 + 24 hpi** | | | **6 + 72 hpi** | | | **24 + 72 hpi** | | |
|  |  | All | 6 | 24 | All | 6 | 72 | All | 24 | 72 |
| 1 | Photosynthesis | 0 |  |  | 1 | ↑ | ↓ | 0 |  |  |
| 2 | Cellular respiration | 0 |  |  | 0 |  |  | 0 |  |  |
| 3 | Carbohydrate metabolism | 0 |  |  | 1 | ↑ | ↓ | 0 |  |  |
| 4 | Amino acid metabolism | 0 |  |  | 1 | ↑ | ↓ | 0 |  |  |
| 5 | Lipid metabolism | 0 |  |  | 2 | ↑↓ | ↓↑ | 0 |  |  |
| 6 | Nucleotide metabolism | 0 |  |  | 0 |  |  | 0 |  |  |
| 7 | Coenzyme metabolism | 0 |  |  | 0 |  |  | 0 |  |  |
| 8 | Polyamine metabolism | 0 |  |  | 0 |  |  | 0 |  |  |
| 9 | Secondary metabolite | 1 | ↑ | ↑ | 0 |  |  | 0 |  |  |
| 10 | Redox homeostasis | 0 |  |  | 0 |  |  | 0 |  |  |
| 11 | Phytohormon action | 1 | ↑ | ↓ | 1 | ↓ | ↑ | 0 |  |  |
| 12 | Chromatin organization | 0 |  |  | 0 |  |  | 0 |  |  |
| 13 | Cell division | 0 |  |  | 0 |  |  | 0 |  |  |
| 14 | DNA damage response | 0 |  |  | 0 |  |  | 0 |  |  |
| 15 | RNA biosynthesis | 0 |  |  | 1 | ↓ | ↑ | 0 |  |  |
| 16 | RNA processing | 0 |  |  | 0 |  |  | 0 |  |  |
| 17 | Protein biosynthesis | 0 |  |  | 0 |  |  | 0 |  |  |
| 18 | Protein modification | 0 |  |  | 0 |  |  | 0 |  |  |
| 19 | Protein homeostasis | 1 | ↑ | ↑ | 0 |  |  | 1 | ↓ | ↓ |
| 20 | Cytoskeleton organization | 0 |  |  | 1 | ↑ | ↓ | 0 |  |  |
| 21 | Cell wall organization | 1 | ↑ | ↑ | 0 |  |  | 0 |  |  |
| 22 | Vesicle trafficking | 0 |  |  | 0 |  |  | 0 |  |  |
| 23 | Protein translocation | 0 |  |  | 0 |  |  | 0 |  |  |
| 24 | Solute transport | 0 |  |  | 2 | ↑↑ | ↓↓ | 0 |  |  |
| 25 | Nutrient uptake | 0 |  |  | 3 | ↑↑↑ | ↓↓↓ | 0 |  |  |
| 26 | External stimuli response | 0 |  |  | 1 | ↑ | ↓ | 0 |  |  |
| 27 | Multi-process regulation | 0 |  |  | 1 | ↓ | ↑ | 0 |  |  |
| 28 | Plant reproduction | 0 |  |  | 0 |  |  | 0 |  |  |
| 30 | clade-specific metabolism | 0 |  |  | 0 |  |  | 0 |  |  |
| 35 | not assigned | 2 | ↓↑ | ↑↑ | 5 | ↓↑↑↑↑ | ↓↓↓↓↓ | 1 | ↑ | ↓ |
| 50 | enzyme classification | 0 |  |  | 0 |  |  | 0 |  |  |
|  | **Total** | 6 |  |  | 20 |  |  | 2 |  |  |
