# Supplementary material for: Aphid-mediated beet yellows virus transmission initiates proviral gene deregulation in sugar beet at early stages of infection
Source: PLoS One. 2024 Oct 1;19(10):e0311368. doi: 10.1371/journal.pone.0311368 (PMC11444407; doi:10.1371/journal.pone.0311368)
Supplement: S5 Table — (DOCX) [file pone.0311368.s007.docx]

| **Suppl. Tab. S5:** Chloroplast-related genes that were solely or commonly identified 6, 24 and 72 hours after BYV inoculation. | | | | |
| --- | --- | --- | --- | --- |
| **gene identifier** | **log_2_ fold change** | | | **putative protein function** |
|  | **6 hpi** | **24 hpi** | **72 hpi** |  |
| \| bv7_176840_wtfh.t1 \| \| --- \| \| bv_007510_faer.t1 \| \| bv4_089990_ssiu.t1 \| \| bv6_148230_nsuc.t1 \| \| bv9_217200_yktd.t1 \| \| bv4_090400_idsy.t1 \| \| bv6_150380_hjur.t1 \| \| bv6_140340_hwtk.t1 \| \| bv6_130960_jfsy.t1 \| \| bv8_189000_week.t1 \| \| bv1_016820_kwie.t1 \| \| bv1_007830_juuw.t1 \| \| bv3_054180_pcrr.t1 \| \| bv2_027280_wuzk.t1 \| \| bv8_196730_uzpt.t1 \| \| bv2_046340_qkot.t1 \| \| bv2_023650_hynn.t1 \| \| bv6_136680_juie.t1 \| \| bv5_102940_qcrz.t1 \| \| bv8_198400_xdmy.t1 \| \| bv5_123200_druz.t1 \| \| bv9_203610_dkoq.t1 \| \| bv6_129050_pnpe.t1 \| \| bv4_081180_zqxf.t1 \| \| bv7_168070_furf.t2 \| \| bv8_193180_ojzz.t1 \| \| bv2_023640_yzom.t1 \| \| bv7_167730_idtj.t1 \| \| bv_008870_gftg.t1 \| \| bv_009200_sruu.t1 \| \| bv4_084680_nwhx.t1 \| \| bv_004050_xzzo.t1 \| \| bv7_159420_rrma.t1 \| \| bv4_087880_iiru.t1 \| \| bv8_186000_dpty.t1 \| \| bv6_146120_wnhu.t1 \| | \| 1,448 \| \| --- \| \| 0,773 \| \| 0,832 \| \| 0,802 \| \| 1,099 \| \| -1,119 \| \| -1,15 \| \| 1,136 \| \| 2,303 \| \| 1,664 \| \| -0,924 \| \| 2,056 \| \| 1,028 \| \| 0,836 \| \| NS \| \| NS \| \| NS \| \| NS \| \| NS \| \| NS \| \| NS \| \| NS \| \| NS \| \| NS \| \| NS \| \| NS \| \| NS \| \| NS \| \| NS \| \| NS \| \| NS \| \| 1,294 \| \| 1,487 \| \| 1,596 \| \| 3,026 \| \| 3,236 \| | \| NS \| \| --- \| \| NS \| \| NS \| \| NS \| \| NS \| \| NS \| \| NS \| \| NS \| \| NS \| \| NS \| \| NS \| \| NS \| \| NS \| \| NS \| \| 3,639 \| \| 1,733 \| \| 3,798 \| \| 3,93 \| \| NS \| \| NS \| \| NS \| \| NS \| \| NS \| \| NS \| \| NS \| \| NS \| \| NS \| \| NS \| \| NS \| \| NS \| \| NS \| \| -2,258 \| \| NS \| \| NS \| \| NS \| \| NS \| | \| NS \| \| --- \| \| NS \| \| NS \| \| NS \| \| NS \| \| NS \| \| NS \| \| NS \| \| NS \| \| NS \| \| NS \| \| NS \| \| NS \| \| NS \| \| NS \| \| NS \| \| NS \| \| NS \| \| -3,045 \| \| -1,076 \| \| -2,121 \| \| -1,677 \| \| -1,271 \| \| -1,127 \| \| 1,595 \| \| -2,055 \| \| -1,678 \| \| -2,57 \| \| -0,943 \| \| -1,247 \| \| -1,508 \| \| NS \| \| -1,827 \| \| -1,66 \| \| -2,15 \| \| -2,041 \| | \| component of PS-II complex \| \| --- \| \| plastidial transcript stability factor \| \| ribosome biogenesis factor mTERF9 \| \| subfamily ABCB transporter \| \| ADP-glucose pyrophosphorylase \| \| beta-amylase \| \| glucose-6-phosphate dehydrogenase \| \| serotonin N-acetyltransferase \| \| glycerophosphodiester phosphodiest. \| \| phosphoglycerate dehydrogenase \| \| ferredoxin nadp reductase \| \| heme-binding protein \| \| protein high chlorophyll fluorescent \| \| protein plastid movement impaired 2 \| \| ferredoxin-1 \| \| alpha-glucan phosphorylase \| \| omega-3 fatty acid desaturase \| \| polyphenol oxidase \| \| early light induced protein \| \| stability factor PPR10 \| \| small heat shock protein \| \| Clp protease adapter protein \| \| pheophytinase \| \| PsbP domain-containing protein \| \| UV-B-induced protein \| \| Arogenate dehydrogenase \| \| omega-3 fatty acid desaturase \| \| phospholipase a1 igamma \| \| uroporphyrinogen III methyltransfer. \| \| quinolinate synthase \| \| phytoene synthase 2 \| \| 9-cis-epoxycarotenoid dioxygenase \| \| alpha carbonic anhydrase 1 \| \| fatty acid desaturase 4 \| \| 5'-adenylylsulfate reductase 1 \| \| ferric reduction oxidase 7 \| |
| NS= not significant | | | | |
